# Patterns and architecture of genomic islands in marine bacteria

**DOI:** 10.1186/1471-2164-13-347

**Published:** 2012-07-29

**Authors:** Beatriz Fernández-Gómez, Antonio Fernàndez-Guerra, Emilio O Casamayor, José M González, Carlos Pedrós-Alió, Silvia G Acinas

**Affiliations:** 1 Department of Marine Biology and Oceanography, Institut de Ciències del Mar, Consejo Superior de Investigaciones Científicas (CSIC), Pg Marítim de la Barceloneta 37-49, ES-08003, Barcelona, Spain; 2 Biogeodynamics & Biodiversity Group, Centre d'Estudis Avançats de Blanes, CEAB-CSIC, ES-17300, Blanes, Spain; 3 Department of Microbiology, University of La Laguna, ES-38206 La Laguna, Tenerife, Spain

**Keywords:** Genomic islands, Horizontal gene transfer, Homologous recombination, Bacterial core genes, Flexible genome, Structure of genomic islands, Patterns within genomic islands, Marine bacteria

## Abstract

**Background:**

Genomic Islands (GIs) have key roles since they modulate the structure and size of bacterial genomes displaying a diverse set of laterally transferred genes. Despite their importance, GIs in marine bacterial genomes have not been explored systematically to uncover possible trends and to analyze their putative ecological significance.

**Results:**

We carried out a comprehensive analysis of GIs in 70 selected marine bacterial genomes detected with IslandViewer to explore the distribution, patterns and functional gene content in these genomic regions. We detected 438 GIs containing a total of 8152 genes. GI number per genome was strongly and positively correlated with the total GI size. In 50% of the genomes analyzed the GIs accounted for approximately 3% of the genome length, with a maximum of 12%. Interestingly, we found transposases particularly enriched within Alphaproteobacteria GIs, and site-specific recombinases in Gammaproteobacteria GIs. We described specific Homologous Recombination GIs (HR-GIs) in several genera of marine Bacteroidetes and in *Shewanella* strains among others. In these HR-GIs, we recurrently found conserved genes such as the β-subunit of DNA-directed RNA polymerase, regulatory sigma factors, the elongation factor Tu and ribosomal protein genes typically associated with the core genome.

**Conclusions:**

Our results indicate that horizontal gene transfer mediated by phages, plasmids and other mobile genetic elements, and HR by site-specific recombinases play important roles in the mobility of clusters of genes between taxa and within closely related genomes, modulating the flexible pool of the genome. Our findings suggest that GIs may increase bacterial fitness under environmental changing conditions by acquiring novel foreign genes and/or modifying gene transcription and/or transduction.

## Background

Bacterial comparative genomics is providing a unique opportunity to retrieve valuable information regarding genome structure, functional diversity and evolution of marine microorganisms. Bacterial genomes are dynamic entities with a conserved pool of genes at the core genome shared at different taxonomic levels, and the flexible (or adaptive) genome, with a number of taxa-specific genes that are not comparable among closely related strains [1,2]. Horizontal gene transfer (HGT) is one of the evolutionary mechanisms enlarging the flexible genome pool of bacterial populations, facilitating their adaptation to new ecological niches [3,4]. GIs are clusters of genes laterally transferred and associated with the flexible genome pool of prokaryotic genomes. These highly variable genome regions have been analyzed for a few bacterial taxa by comparative genome analysis [1]. In well-known marine bacteria such as *Prochlorococcus*, *Synechococcus,* and *Shewanella* comparative genome analysis has revealed a substantial number of species-specific genes [5-7]. Those studies revealed an unsaturated pangenome size, reflecting the existence of new lineages and the heterogeneity among the flexible genome pool.

Species-specific genes are commonly found in GIs [1,8]. These are important genomic regions causing significant genetic differences between closely related taxa, and they may reveal particular ecologically relevant features of the genomes [9,10] and virus-bacteria interaction [11]. GIs may harbor a large set of genes with different origins. However, using a hypothesis-free approach for the identification of GIs some common features can be recognized suggesting that GIs could be perceived as a superfamily of mobile elements [12]. Genes found within GIs are very diverse: from key genes for survival in specific environments to virulence, and/or antibiotic resistance genes. In fact, GIs enriched in virulence genes and in Clustered Regularly Interspaced Palindromic Repeats (CRISPR) confer resistance to exogenous genetic elements such as plasmids and phages [13]. Thus, GI content may hold clues about the lifestyle or survival strategies of bacteria [14]. Another common characteristic of the GIs is the enrichment in novel genes without any orthologous groups detected in the Clusters of Orthologous Groups (COG) database or any other known functional gene families [15].

Extensive literature exists on GIs in pathogenic bacterial strains (referred to as pathogenic islands) where their relevance is known for antibiotic resistance or virulence stages [1,16,17]. In environmental microorganisms GIs have been associated with the presence of catabolic pathways for organic pollutants, thus conferring adaptive traits in some *Pseudomonas* strains [18]. Another ecological feature associated with GIs is the presence of genes for magnetite biomineralization in what is called the magnetosome island in the alphaproteobacterium *Magnetospirillum gryphiswaldense*[19], or secondary metabolism in marine *Actinobacteria* strains [20]. Another case is the acquisition of a capsular polysaccharide biosynthesis gene cluster by the non-pathogenic soil inhabitant *Burkholderia thailandensis* with similar characteristics to the virulence gene cluster of the pathogenic *Burkholderia pseudomallei* (responsible for the melioidosis disease) [21]. In Cyanobacteria, GIs from several strains of *Prochlorococcus marinus*[5,10] and *Synechococcus* strains [6] have been reported. Also, the GIs of two freshwater filamentous toxin-producing cyanobacteria were found with diverse comparative approaches [22]. For Gammaproteobacteria, GIs were described for the high pressure adapted *Photobacterium profundum* SS9 strain [23], the marine coastal *Vibrio vulnificus*[24], *Alteromonas macleodii*[25] and *Shewanella baltica* strains [26]. In Alphaproteobacteria, GIs were found in SAR11 (Candidatus *Pelagibacter ubique* strain HTCC1062) referred to as hypervariable regions in the original study [27]. In aquatic Bacteroidetes GIs were described in *Salinibacter ruber*, a very abundant bacterium in solar salterns [28]. Finally, virulence genes of typical pathogenic island were reported in marine bacteria genomes in a comparative study [29].

However, GIs in marine bacterial genomes have not been explored systematically and a comparative analysis is still lacking. Bacteria can adapt to different light regimes [30] or to attach to organic matter particles [31] for example, allowing niche differentiation and coexistence of different species. Therefore, analyses of GIs of marine bacterial genomes may reveal genes for adaptation to different ecological niches. In this study, we carried out a comprehensive analysis of GIs in 70 selected marine bacterial genomes that represented abundant and ecologically relevant bacteria in the ocean. We assembled a database of 8152 genes found in GIs of marine bacteria and screened it for possible patterns and clues about the ecological relevance of GIs in marine bacteria.

## Results and discussion

### Accuracy of GI prediction: previous (control) vs. GIs detected in this study

**Figure 1:**
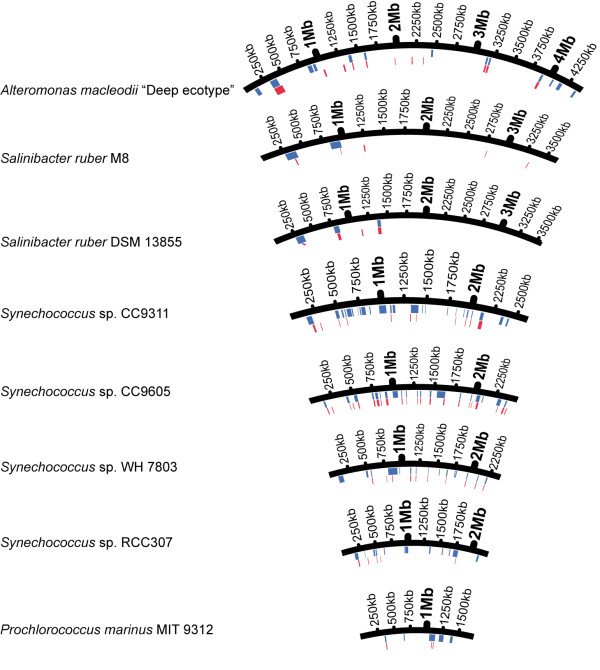
**Positions of the GIs in eight selected marine bacterial genomes used as controls.** Blue bars show the GIs available in previous studies and red bars show the GIs predicted by IslandViewer. This graphic includes all GIs detected by IslandViewer although only GIs > 9.5 kb were included in our dataset for further analyses.

It is impractical and excessively time consuming to manually curate 70 genomes in order to identify all the GIs in them. IslandViewer has been shown to be one of the best tools for this purpose [32]. IslandViewer is a web-based interface that integrates several methods for identification and visualization of GIs: IslandPick [33], SIGI-HMM [34] and IslandPath-DIMOB [35]. The GIs prediction tools integrated in IslandViewer were validated by Langille et al., 2008 [33] using a reference database of 675 genomes and based on comparisons with at least three closely related genomes. The existence of well-annotated genomes that are close phylogenetic relatives of the examined genome is usually not a problem for pathogenic bacteria. However, for marine bacteria there are much less genomes sequenced and even less have been manually curated. We tried to have at least three closely related genomes for each marine genome examined, but this was not always possible. For this reason, we decided to test: (i) the number of GIs detected by IslandViewer compared to those GIs detected in a few representative marine genomes that had been manually annotated and published and (ii) potential differences in functional annotation between the GIs detected by both approaches. In many cases GIs detected in previous studies were based on one approach only: either comparison of two genomes or differences in the tetranucleotide frequency and occurrence of mobility genes (Table 1). IslandViewer seeks several characteristics and, therefore, we can expect a more robust and more conservative detection of GIs. The genomes used as controls are shown in Figure 1 and Table 1. 

**Table 1:** Comparison of the GIs of eight marine bacteria referred to as Control Genomes where GIs were available in previous studies and the GIs predicted by this study for the same genomes by IslandViewer

**Bacterial strain**	**Previous studies GIs**		**IslandViewer GIs**				**Comparison analyses (Previous vs. IslandViewer GIs)**		
	**Number of GIs**	**Total GIs length detected in control genomes (kb)**	**#GIs present (+)**	**Extra GIs**^**b**^**(+)**	**#GIs absent (−)**	Total GIs length with IslandViewer prediction (kb)*	**(% overlap)**^**a**^**(kb)**	**Recall**^**b**^**(sensitivity)(%)**	**Precision**^**c**^**(%)**
*P. marinus* str. MIT9312^**1**^	5	233	3^d^	0	3	44.9	19.3	50	100
*S.* sp. RCC307^**2**^	15	271	9	1	6	56.9	18.7	60	90
*S.* sp. WH7803^**2**^	11	344	3	2	8	73.7	20.3	27	60
*S.* sp. CC9605^**2**^	20	505.3	18	4	2	300.4	54.3	90	82
*S.* sp. CC9311^**2**^	24	578.6	9	2	15	125.9	16.9	38	82
*A. macleodii* “deep ecotype”^**3**^	13	480	6	5	7	272.7	69.6	46	55
*S. ruber* DSM13855^**4**^	3	221.8	3	1	0	168.2	41.1	100	75
*S. ruber* M8^**4**^	2	265.9	2	3	0	144.9	44.5	100	40
**Average**							**35.58**	**64**	**73**

^**1**^Coleman *et al*., 2006. Method used to detect GIs: Comparative genomics using 2 *Prochlorococcus marinus* genomes.

^**2**^Dufresne *et al*., 2008. Method used to detect GIs in *Synechococcus* genomes: Modified protocols published by Hsiao [15] and Rusch et al. [36] specifically deviations of tetranucleotide frequency, presence of MGE, tRNAs and the occurrence of core genome gene blocks.

^**3**^Ivars-Martínez *et al*., 2008. Method used to detect GIs: Comparative genomics using 2 *Alteromonas macleodii* strains genomes.

^**4**^Peña *et al*., 2010. Method used to detect GIs: Comparative genomics using 2 *Salinibacter ruber* genomes.

a)% overlap between GIs of previous studies and this study.

b) Recall (sensitivity): GIs present (+)/ [GIs present (+) plus GIs absent (−)].

c) Precision: GIs present (+)/[GIs present (+) plus Extra GIs (+)].

d) Two of these three GIs detected by IslandViewer match different sections of the biggest GI of *Prochlorococcus marinus* str. MIT9312 (see in Figure 1)*.*

* All GIs detected by IslandViewer/IslandPick included some small genomic islets (<9.5 kb) that are not included in Additional file 3: Table S3.

The GIs of previous studies are shown in blue in Figure 1 together with the GIs detected by IslandViewer in red. The percentage of GIs detected ranged from 27% in *Synechococcus* RCC307 to 100% in both *Salinibacter* strains. Another discrepancy was that some areas were detected as GIs that were not annotated as such in the genomes. In most cases there were only one or two “extra GIs” per genome, although there were five in *Alteromonas* and four in *Synechococcus* CC9605. In all cases, however, these extra GIs were very short (Figure 1), usually smaller than 8 kb, and therefore these GIs were not included in our final dataset. In terms of the length of DNA in GIs (in kb) IslandViewer detected approximately 20–60% of the length in manually annotated GIs with an average of 36% (Table 1). These percentages were smaller than those found in a previous test based on 118 bacterial genomes (mostly pathogenic bacteria with many closely related genomes available) also using IslandViewer (88% on average) [32,33]. However, both precision (average 73%) and sensitivity (average 64%) were very good when compared with the different methods explored in [33] (see their Table 1).

Additionally, we compared the functional gene annotation in the GIs that were detected by both systems (previous studies and this study; Table 1). We did two types of comparisons. In the first one, we considered each genome separately and, in the second one, we considered all the control genomes pooled together. Obviously, for this purpose, only genes with clearly assigned gene categories (GO) could be considered. Thus, out of all the detected genes, the HPs genes were discarded for this comparison (Additional file 1). The number of genes ranged from 51 to 225 in the manually annotated subset and from 16 to 116 in the automatically annotated subset. Fischer’s exact tests revealed no significant differences in the proportion of genes in each GO category between the two data subsets. Finally, we pooled all the annotated genes in previous published GIs of the eight control genomes (1065 genes), and all the predicted genes in the GIs for the same genomes (397 genes) detected by IslandViewer. We only found three specific GO terms with significantly different distributions in both datasets. One GO term related to photosynthesis (GO:0015979) was found underrepresented in the IslandViewer annotated database. This was probably due to the smaller percentage of GIs detected in some of the cyanobacterial strains, such as *Synechococcus* sp. CC9311 and RCC307 or *Prochlorococcus marinus* MIT9312. Two other GO terms were overrepresented in the IslandViewer annotated data set. These were related to DNA recombination (GO:0006310) and DNA binding (GO:003677). This was likely due to the fact that IslandViewer detects many GIs based on the existence of mobile genes (Additional file 2). Likely, this was also the cause of the “extra GIs”, since isolated mobile elements tend to be ignored during manual annotation unless they are an important objective for the researcher (and usually they are not). Thus, IslandViewer should be more efficient at detecting these GIs than previous studies based on a single approach.

In conclusion, taking into account that our GIs prediction is not exhaustive and will be missing some of the true GIs, the important conclusion for the present work is that the functional analyses of genes in GIs from a large number of marine bacterial genomes will uncover valid patterns and ecologically relevant information in this flexible genome pool.

### Quantitative importance of GIs in marine bacterial genomes

The 70 selected marine bacterial genomes represent the four major prokaryotic taxa in the ocean: Cyanobacteria (16 genomes), Gammaproteobacteria (17), Alphaproteobacteria (16) and Bacteroidetes (21) (Additional file 3: Table S3). These four bacterial taxa account for up to 80% of the total marine bacterioplankton [36]. Bacteroidetes genomes included 14 Flavobacteria and 7 non-marine Bacteroidetes (*Bacteroides* spp.) used as out-groups. Several genomes of closely related bacterial strains from each phylogenetic group were included to investigate the rate of variability of GIs at intra-specific level and explore their relevance as main contributors to strain-specific genes. IslandViewer detects GIs ≥8 kb although only those ≥9.5 kb were used to compile our database for further analyses to be consistent with previous published studies [6,9,25,37].

**Figure 2:**
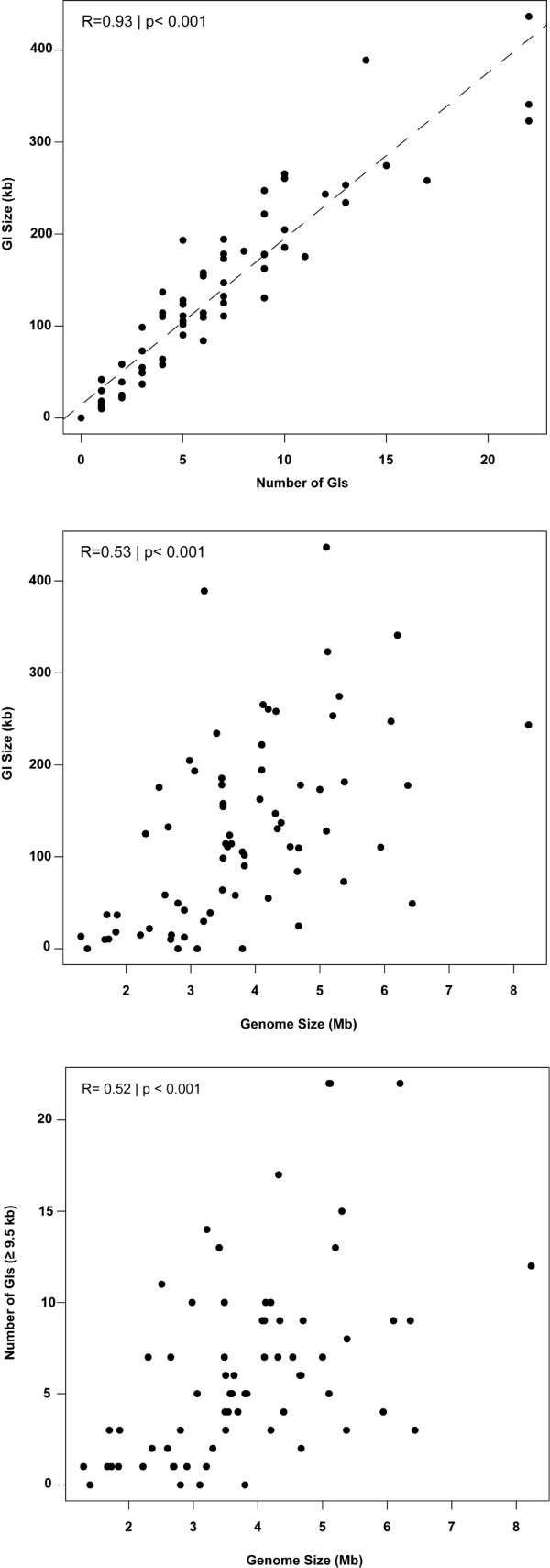
**Patterns of GIs in marine bacterial genomes.A**) Relationship between number of GIs per bacterial genome with GI size (in kb).** B**) Relationship between bacterial genome and GI size and **C**) relationship between genome size and number of GIs (≥9.5 kb).

GIs were detected in 66 out of the 70 bacterial genomes (Additional file 2 and Additional file 3). No GIs were detected in the genomes of *Pelagibacter ubique* HTCC1062 and the marine Bacteroidetes Flavobacteria BBFL7, Flavobacteria ALC-1, and *Flavobacterium psychrophilum* JIP02/86. The absence of GIs in these genomes may be related to the small genome size of some of them (between 1.4 and 3.8 Mb), as well as to the lack of sensitivity of the GI predictor when suitable genomes were not available for comparison. Overall, we detected 438 GIs spanning a total of 8.87 Mb (Additional file 3). The size of individual GIs ranged from 10 to 436 kb (389 kb per genome on average, considering the marine genomes only). As expected, the total GI size was strongly and positively correlated with the number of GIs per genome (R = 0.93, p <0.001) (Figure 2A). Significant (p <0.001) but moderate correlations (R = 0.53 and 0.52, respectively) were observed between genome size and both GI size and number of GIs per genome (Figure 2B and 2C). When the data were analyzed separately for each of the four classes, Cyanobacteria (R = 0.60, p < 0.05) and Bacteroidetes (R = 0.78, p <0.001) showed significant correlations (Additional file 4, panels A and D) while the Proteobacteria did not (Additional file 4, panels B and C). For picocyanobacteria, stronger correlations (R^2^ = 0.9) were observed between GIs size and genome size in 14 genomes from the two ecologically important genera *Synechococcus* and *Prochlorococcus*[6]. In our case, we included 16 cyanobacteria genomes from six different genera and therefore we observed higher variability with lower correlations (but still significant). 

**Figure 3:**
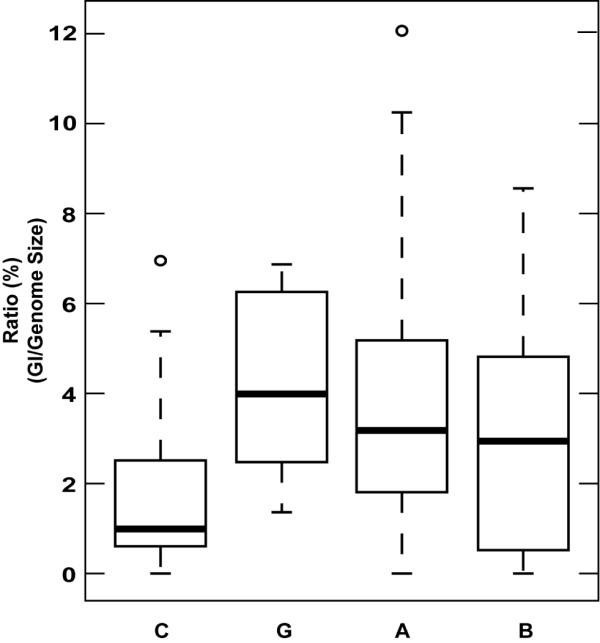
**Box- and whiskers graphic of the GI ratio (in%) for the 70 marine bacteria.** Genomes are ranked from highest (12%) to lowest (0%) and grouped in 4 main phylogenetic affiliation represented as follows: C (Cyanobacteria), G (Gammaproteobacteria), A (Alphaproteobacteria) and B (Bacteroidetes). The graph shows the median (thick horizontal line), the upper and lower quartile (rectangle), the maximum and minimum values excluding outliers (discontinuous line), and finally circle represents an outlier.

For any given range of genome sizes, there was a large variability in the length of GIs (Additional file 3). The fraction of the bacterial genome represented by GIs ranged from 0 to 12% (Figure 3). Most genomes showed a ratio between 2 and 5%. The Cyanobacteria showed a significantly lower average ratio than the other three classes. However, this is likely due to the low GI detection rate of IslandViewer in the case of several Cyanobacteria (Table 1). Otherwise, there were no significant differences among classes, although the Gammaproteobacteria showed a lower variability than Bacteroidetes and Alphaproteobacteria (Figure 3). The genomes with the highest ratios for each main bacterial class were the alphaproteobacterium *Rhodobacter sphaeroides* ATCC1705 (12%), the cyanobacterium *Synechococcus* sp. CC9605 (7%), the gammaproteobacterium *Psychrobacter* sp. PRwf-1 (6.8%), and the flavobacterium *Robiginitalea biformata* HTCC2501 (4.5%; Additional file 3). In a previous study, *Synechococcus* GIs were shown to be between 10 and 31% of their genomes [6] and similar percentages, up to 17%, have been found also for pathogenic islands in *Escherichia coli*[38]. All the marine bacteria examined have a lower percentage of their genome in GIs. 

Interestingly, high intra-specific variability in GIs size was observed in some members of each group. Two *Synechococcus* strains, for example, with genomes of 2.2 and 2.6 Mb respectively, showed very different GI ratios (15 and 175 kb respectively). Similarly, small differences in genome size between two *Shewanella baltica* strains (MR-4 and OS155, with 4.7 and 5.12 Mb respectively) contrasted with marked differences in their GIs ratios (3.7 and 6.3% respectively) (Additional file 3 and Additional file 4).

### Architecture of marine bacterial GIs

As a common characteristic we found that 70% of the detected GIs contained MGE, mostly transposases, conjugative transposons, integrons or phage integrase-related genes in accordance with previous GIs studies [39,40]. In addition, we observed that at least 27% of the GIs were flanked by or contained tRNAs, probably acting as the integration sites for GIs [41,42].

**Figure 4:**
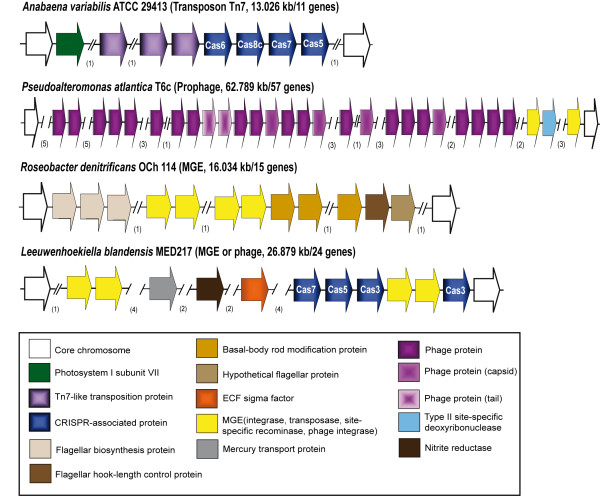
**Structure (5´-3´) of the Horizontal Gene Transfer (HGT)-GIs representative of four marine bacteria.** Genomes belonged to Cyanobacteria, Gammaproteobacteria, Alphaproteobacteria and marine Bacteroidetes. The hypothetical origin of HGT (via prophage, transposon or other MGE), the length of the GI (in kb) and the number of genes integrated are shown in brackets. Colors indicate the variety of genes observed within the GIs. Numbers in brackets under the genes indicate the HPs and other genes not considered for the figure.

Most GIs in marine bacterial genomes could be assigned to one of the two following architectures. First, many of these GIs exhibited a high content of HP, as well as different sets of genes, suggesting that they had originated through horizontal gene transfer by phages, conjugative transposons or other MGEs. From now on, we will refer to these GIs as HGT-GIs. One example from each bacterial class examined is shown in Figure 4. The cyanobacterium *Anabaena variabilis* ATCC 29413 displayed a gene related to *psaC* of the photosystem I subunit VII, four *cas* genes related to CRISPR system and three Tn7-like transposition genes in a single GI of 13 kb. The gammaproteobacterium *Pseudoalteromonas atlantica* T6 presented a GI of 62.7 kb mostly constituted by a prophage with many phage related genes. Also, in the alphaproteobacterium *Roseobacter denitrificans* OCh 114 we detected a GI of 16 kb with many flagellar protein genes and MGE elements. Finally, the marine Bacteroidetes *Leeuwenhoekiella blandensis* MED217 had a GI of 26.8 kb with multiple genes of MGE, *cas*, and a nitrite reductase gene. Among the marine Bacteroidetes, this gene has been only found in the deep sea flavobacterium *Zunongwangia profunda* SM-A87 with capacity to hydrolyze organic nitrogen [43]. Many other GIs presented ecologically interesting genes but specific details for each one are out of the scope of this article. 

**Figure 5:**
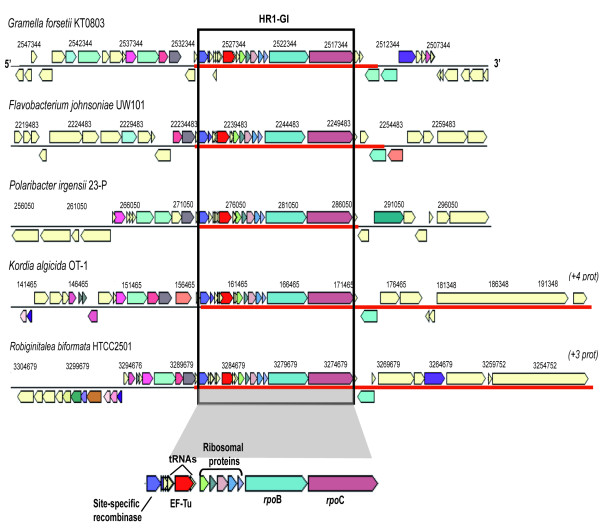
**Structure (5´-3´) of the Homologous Recombination GIs (HR-GIs) in marine bacterial genomes.** HR1-GI detected in five different genera of marine Bacteroidetes. The synteny and the gene cassette shared by these genomes are within the black box. Red line indicates the length of the GI detected which varied among the genomes.

And secondly, we found many GIs that contained site-specific recombinases and tRNAs. Interestingly, these GIs harbor many core genes, almost no HPs, and had a structure that could be repeatedly detected in closely related strains but also in different genera (Figure 5). We hypothesized that these genomic fragments were likely transferred via HR. We will refer to these as HR-GIs. Quite likely these genomic cassettes may be also mobilized within the same genome by the transposases that some of them have at their flanks. Flavobacteria was one of the classes with more conspicuous HR-GIs. Figure 5 shows an example of one of the HR-GI named as HR1-GI. This GI was found in a high number of Bacteroidetes (Additional file 5) of which five were specifically detected in our dataset and are shown in Figure 5 as an example. Despite differences in the total length of the island (from 15.9 to 44 kb), synteny was maintained for a cassette consisting of a substantial number of genes (see black rectangle in Figure 5). It is notorious that the genes observed upstream of the cassette were quite different in every genome (Figure 5). Surprisingly, conserved genes than encode products as important as the β-subunit of DNA-directed RNA polymerase, the elongation factor Tu, sigma factors, transcription termination factors, and ribosomal proteins were recurrently detected in these HR-GIs, and they also contained site-specific recombinases and tRNAs. The HR1-GI detected in five marine flavobacteria (Figure 5) was further examined in other Bacteroidetes representatives. We observed identical synteny in nine flavobacteria, three sphingobacteria and three *Bacteroides* (Additional file 5). The only variants were a few gene insertions in *Capnocytophaga ochracea* DSM 7271 and some deletions in sphingobacterial genomes. This particular HR1-GI, therefore, was well conserved throughout the Bacteroidetes phylum.

Some of the genes present in these particular HR-GIs encode ribosomal proteins, which are known to be highly expressed, usually with a sequence composition different from the rest of the genome [44]. As a consequence, these genome fragments might appear as false-positive predictions of GIs if sequence composition bias (% GC content) were used as the only criterion to identify GIs. IslandViewer integrated the three most accurate GI prediction programs [15,32,45], each using different approaches to predict GIs and, in effect, both HR-GIs were detected by more than one tool (see rows in yellow in Additional file 2). However, we looked for additional evidence that these gene-cassettes were not false positives, that is, that they were in a true GI.

For this purpose, phylogenetic trees were reconstructed with sequences from 20 Bacteroidetes genomes based on RpoB and EF-Tu gene sequences, which are found in HR1-GIs, as well as the 16S rRNA. If these GIs were false-positives, we would expect the phylogenies of RpoB and EF-Tu genes to match that of 16S rRNA. If these were true GIs subject to HR, however, we would expect somewhat different phylogenies for 16S rRNA, and RpoB and EF-Tu genes (Additional file 6). Although the general topology among Flavobacteria, Sphingobacteria and *Bacteroides* branches was conserved with the three genes, several discrepancies could be detected between the 16S rRNA phylogeny and those of the other two genes **(**see black triangles and circles in Additional file 6**)**. This is in accordance with the two functional genes following similar evolutionary trends and belonging to a GI.

**Figure 6:**
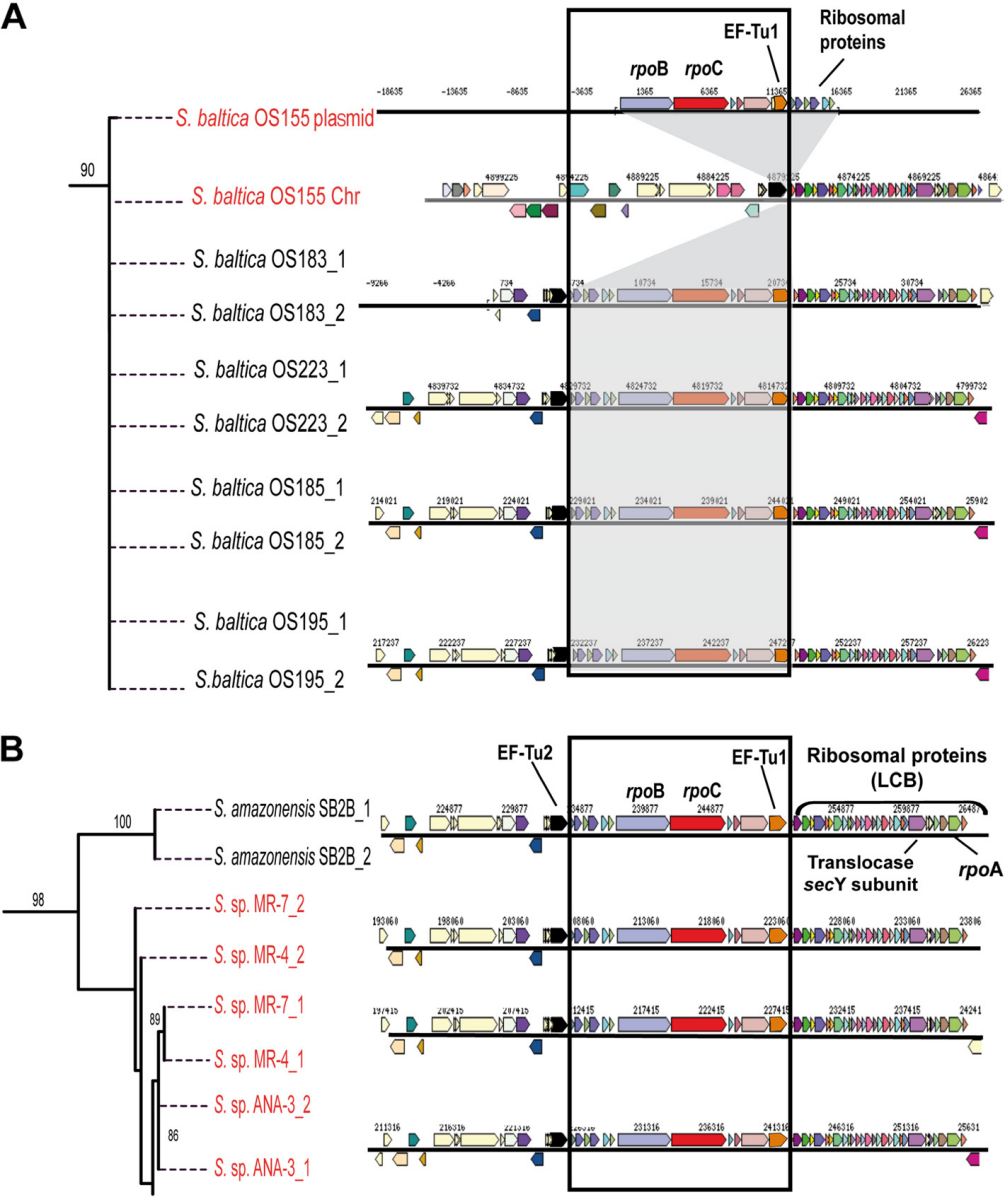
**Zoom of two sections of the phylogenic tree of the EF-Tu gene in *Shewanella* strains.** The completed phylogenetic tree shows the two gene copies of the EF-Tu of 19 *Shewanella* strains where HR1-GI was observed marked as a black box (see Figure 4SM). **A**) The insertion location of the plasmid pSbal03 of *Shewanella baltica* OS155 in its chromosome (both labeled in red) is shown in grey and the most representative genes are indicated within HR1-GI. **B**) *Shewanella* strains labeled in red show discrepancies in the EF-Tu phylogeny when both EF-Tu genes were compared.

A second line of evidence in support of the HR1-GI being true islands comes from a plasmid found in *Shewanella baltica* OS155 (pSbal03), containing the same gene-cassette of HR1-GI (except for one gene). The genes in this cassette were absent from the corresponding chromosome, further showing that HR1-GI is in fact laterally transferred (Figure 6A). Interestingly, identical gene structure to this plasmid with a translocation of six ribosomal proteins was observed in 20 other *Shewanella* strains (Figure 6A). Plasmid integration in the host chromosome by HR is a well known phenomenon in bacteria such as *E. coli*, *Bacillus subtilis*, *Enterococcus faecalis* and others [46]. Usually, the site of integration in the genome corresponds to the chromosomal location of the fragment shared with the plasmid. In our case, the hypothetical insertion of the plasmid in the *Shewanella baltica* OS155 chromosome is located next to the chromosomal EF-Tu gene shared by the plasmid and next to a large cluster of 15 ribosomal proteins and *rpoA* (Figure 6A). This cluster of ribosomal protein genes is considered to be a locally collinear block (LCB) meaning a contiguous segment of genes with low rearrangements [47]. The fact that most of the *Shewanella* strains harbor two copies of EF-Tu genes fits with the idea of one of them belonging to this or a similar *Shewanella* plasmid. We conducted phylogenetic reconstruction of EF-Tu genes in these 19 *Shewanella* strains, revealing certain anomalies in the tree topology (Additional file 7). For instance, both EF-Tu gene copies of *Shewanella* sp. MR-4 and MR-7, two isolates retrieved from different depths of the Black Sea [48], clustered with each other instead of with the EF-Tu gene copy of their genome (Figure 6B). This finding is consistent with a recent study in which high level of HR has been discovered among co-occurring *Shewanella baltica* isolates [26]. It is known that recombination plays a cohesive role in bacteria within closely related lineages, because HR is rare between distant phylogenetic taxa [26,49,50]. However, HR and other mechanisms such as genomic rearrangements have been also identified as a key role driving speciation in several aquatic bacterial populations [51-54]. Our findings for *Shewanella* strains seems to indicate that this HR1-GI was first integrated into the *Shewanella baltica* OS155 chromosome via plasmid and later transferred to other co-existing strains by HR. We have observed identical HR-GIs not only within strains of the same species but also across genera in Bacteroidetes (Figure 5). 

These HR1-GIs related to transcription and its regulation and translation processes if they have been adequately integrated might be beneficial under particular conditions. We observed this HR1-GI next to a cluster of ribosomal protein genes and *rpoA* as occurs for many *Shewanella* strains or next to the TonB-dependent receptors as is the case for some marine Bacteroidetes (data not shown). This strategic location may favor an increased level of synthesis of proteins required at critical moments or at transitions to different lifestyles, as suggested for marine Bacteroidetes [31].

### Functional annotation of the prokaryotic GIs

**Figure 7:**
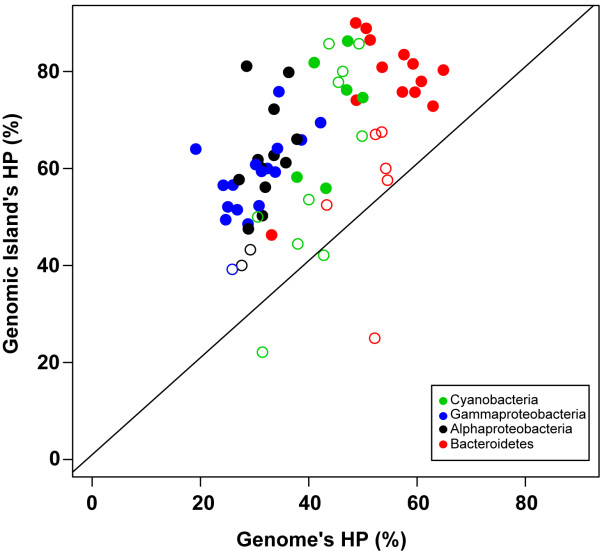
**Percentage of HPs within the GIs and on average for each bacterial genome.** Each bacterial taxon is represented by different colors. Solid circles show statistically significant differences using the corrected p-value (Bonferroni) < 0.05 after Fisher Exact Test, while empty circles did not show significant differences.

One of the reported features of prokaryotic GIs is a higher ratio of genes encoding HPs than in other genome regions [15]. Indeed, we found significantly higher percentage of HPs within GIs than the average for the whole genome in 71% of the genomes (Fisher’s test with the Bonferroni correction: p <0.05) (Figure 7). The 19 genomes with non-significant differences of HP within and outside the GIs were mostly marine Bacteroidetes or Cyanobacteria (Figure 7). These two bacterial classes were the ones with largest % HP in their genomes (Figure 7). We believe this is due to the lower number of well-studied strains compared to Proteobacteria. Thus, the% HP is larger throughout the genome and therefore no significant differences were found within and outside GIs. On average 55–60% of the genes in GIs encode HPs. In this respect, the GIs of marine bacteria are like those described before in pathogenic bacteria, where HPs constituted 53% of the genes within GIs versus 28% in the rest of the genome [15]. 

**Figure 8:**
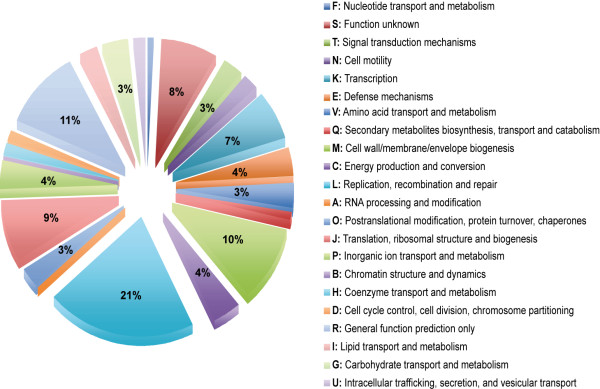
**Distribution of annotated genes within the GIs according to their COG category.** Percentage shown for those categories accounting for ≥3%.

Next, we annotated the genes within GIs by assigning them to functional categories with two approaches: Clusters of Orthologous Groups (COG) and GeneOntology (GO) with a total of 3725 and 3360 genes respectively to which a function could be assigned. Their distribution in the 22 COG categories appears in Figure 8. As expected category L (replication, recombination, and repair) contained over 20% of the total in agreement with the high proportion of transposases, integrases, and recombinase-related genes found in GIs. The next category was R, "general prediction only" with 11%. This basically includes proteins for which a more specific function could not be assigned and therefore it is not informative. Cell wall/membrane/envelope biogenesis (M) with 10% and the translation/ribosomal structure and biogenesis (J) with 9% were especially well represented. Cell motility (N), defense mechanisms (E), and inorganic ion transport and metabolism (P) were also represented (3–4%).

**Figure 9:**
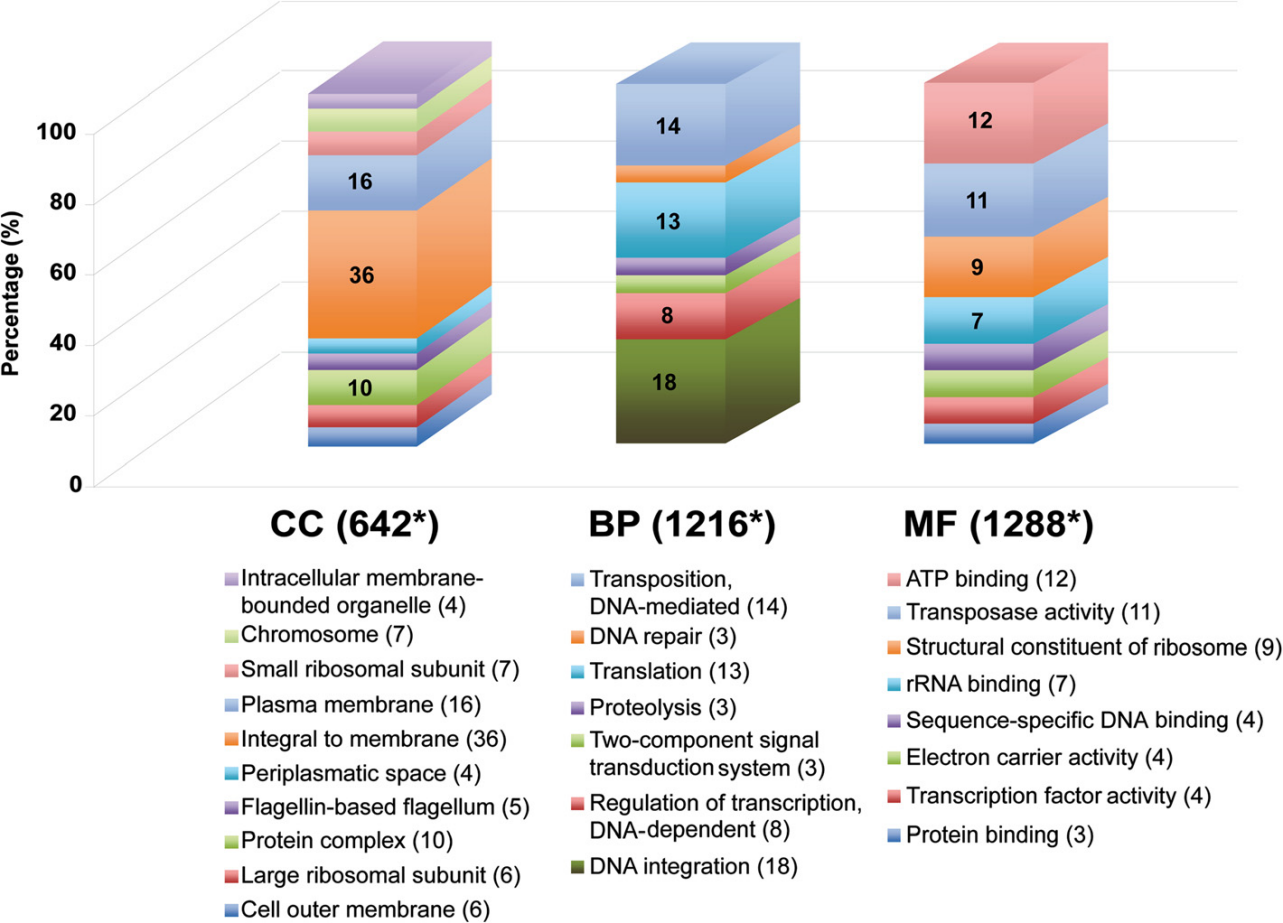
**Distribution of annotated genes within the GIs according to GO classification.** Functional categories were split in three: Cellular Components (CC), Biological Processes (BP) and Molecular Functions (MF). Asterisk means the number of annotated genes to each main category. Numbers between parentheses indicate the percentage of appearance (only shown if ≥3%).

In addition, we used Blast2GO to determine the functional annotation based on GO terms into the three major functional categories: Cellular Component (CC), Biological Process (BP) and Molecular Function (MF) (Figure 9). CC category genes were very abundant especially those associated with the plasma membrane specifically (16%) or with membranes in general (36%). In the BP category, DNA integration (18%) and transposition DNA-mediated genes (14%) were abundant as expected. We found 13% of the genes were associated with translation. Other genes related to the two-component signal transduction system, and DNA repair and proteolysis related proteins with 3% each were frequent. In the MF category, the most abundant were the ATP binding (12%) and transposase activity (11%). Structural constituents of ribosomes (9%) including ribosomal proteins from small and large subunits (7 and 6%) were significant. Flagellin proteins (5%) were also frequent (Figure 9). In summary, both functional annotation approaches revealed that a diverse range of biologically relevant genes were present in the GIs. As expected, these included genes for mobility of DNA fragments but, interestingly, also genes associated with basic cellular mechanisms such as translation and regulation of transcription and transduction.

The possibility to move clusters of genes associated with transcription and their regulation (presence of β subunit of DNA-directed RNA polymerases, elongation factor Tu, sigma factors, transcription termination factors) and translation between closely related genomes and/or different genera may be beneficial for bacterial fitness under changing environmental conditions when an increased level of the transcription and synthesis of certain proteins may be needed.

It seems that lateral transfer genes related to protein-protein interactions may take up several million of years to be established into the regulatory network of the host. Thus, the gene clusters detected in GIs may represent ancient transfer events [55]. If they have been integrated next to other related regulatory proteins or transcriptional factors, their selection might have been favored [55]. Additionally, IS/transposase genes located nearby and within GIs may have a key role to activate transcription of those genes by either introducing complete or partial promoters located within the element itself, by disrupting another gene that may inhibit transcription [56] or by inserting foreign genes into positions where they become regulated by endogenous promoters [57].

### Differences in GI gene content among marine bacterial classes

To find out whether there were differences in the functional categories found within GIs of the four different bacterial classes, we compared the representation of GO terms in each main phylogenetic group with the remaining dataset by paired Fisher´s Exact Tests (Additional file 8). There were significant differences in all cases, indicating that each bacterial class had a different set of functions preferentially represented in their GIs. Our results have to be interpreted with caution since differences in GIs number between organisms might partly be due to variations in efficiency of GI prediction between organisms, due for instance to other factors causing a bias in sequence composition such as a difference in gene expression level [58].

The GO terms that were significantly overrepresented (p-value <0.01 after False Discovery Rate correction) in each of the four phylogenetic groups are listed in Table 2. Cyanobacteria were, by far, the taxon with highest and most diverse number of enriched GO terms (a total of 18). Most of these were related to photosynthesis, both to the antenna or photosystem proteins and to the electron transport system. These photosynthetic related genes (a total of 30 genes) were found within GIs in 50% of the Cyanobacteria genomes, suggesting that they are a rather common feature among Cyanobacteria GIs. Other GO terms enriched in Cyanobacteria were linked to proteolysis/hydrolysis activity, glucose metabolism, histidine and cobalamin biosynthesis.

**Table 2:** Gene Ontology (GO) terms enrichment analyses of GIs in 4 main phylogenetic groups

**Taxonomic group**	**GO term**	**Name**	**Ontology category**	**FDR**	**FWER**	**Single test p-value**
**Cyanobacteria**	GO:0015979	Photosynthesis	BP	8.3E-7	4.1E-7	***
	GO:0030089	Phycobilisome	CC	3.0E-6	3.2E-6	***
	GO:0009898	Internal side of plasma membrane	CC	3.0E-6	3.7E-6	***
	GO:0018298	Protein-chromophore linkage	BP	7.0E-4	2.3E-3	***
	GO:0009521	Photosystem	CC	7.0E-4	2.3E-3	***
	GO:0046914	Transition metal ion binding	MF	1.7E-3	6.7E-3	***
	GO:0004175	Endopeptidase activity	MF	1.8E-3	8.5E-3	***
	GO:0006508	Proteolysis	BP	3.3E-3	3.0E-2	***
	GO:0016740	Transferase activity	MF	3.3E-3	3.0E-2	***
	GO:0009236	Cobalamin biosynthetic process	BP	3.4E-3	3.3E-2	***
	GO:0022900	Electron transport chain	BP	4.5E-3	5.0E-2	***
	GO:0006006	Glucose metabolic process	BP	5.0E-3	5.6E-2	***
	GO:0033178	Proton-transporting two-sector ATPase complex	CC	6.1E-3	9.3E-2	**
	GO:0042777	Plasma membrane ATP synthesis coupled proton transport	BP	6.1E-3	9.3E-2	**
	GO:0046933	Hydrogen ion transporting ATP synthase activity	MF	6.1E-3	9.3E-2	**
	GO:0000105	Histidine biosynthetic process	BP	6.1E-3	9.3E-2	**
	GO:0043231	Intracellular membrane-bounded organelle	MF	6.1E-3	9.8E-2	**
	GO:0016820	Hydrolase activity, acting on acid anhydrides	MF	7.2E-3	1.2E-1	**
**Gammaprotebacteria**	GO:0009009	Site-specific recombinase activity	MF	7.3E-4	1.8E-4	***
	GO:0016874	Ligase activity	MF	7.4E-4	3.7E-4	***
**Alphaproteobacteria**	GO:0042255	Ribosome assembly	BP	3.3E-9	5.0E-9	***
	GO:0003964	RNA-directed DNA polymerase activity	MF	3.6E-4	9.1E-4	***
	GO:0003995	Acyl-CoA dehydrogenase activity	MF	4.1E-4	1.1E-3	***
	GO:0040011	Locomotion	BP	7.6E-4	2.3E-3	***
	GO:0004803	Transposase activity	MF	1.2E-3	3.8E-3	***
	GO:0006313	Transposition, DNA-mediated	BP	5.5E-3	2.0E-2	***
**Flavobacteria**	GO:0015662	ATPase activity, coupled to transmembrane	MF	1.8E-3	1.4E-3	***
		movement of ions				
	GO:0003899	DNA-directed RNA polymerase activity	MF	2.2E-3	2.9E-3	***
	GO:0003711	Transcription elongation regulator activity	MF	2.2E-3	1.0E-2	***
	GO:0032968	Positive regulation of RNA elongation from	BP	2.2E-3	1.0E-2	***
		RNA pol. II promoter				
	GO:0003924	GTPase activity	MF	3.5E-3	1.8E-2	***
	GO:0032549	Ribonucleoside binding	MF	3.7E-3	2.4E-2	***
	GO:0031564	Transcription antitermination	BP	3.7E-3	2.4E-2	***
	GO:0008135	Translation factor activity, nucleic acid binding	MF	5.0E-3	3.6E-2	***
**Non-**	GO:0003735	Structural constituent of ribosome	MF	1.3E-9	5.3E-9	***
**Marine Bacteroidetes**	GO:0006412	Translation	BP	1.3E-9	5.3E-9	***
	GO:0019843	rRNA binding	MF	1.3E-9	5.3E-9	***
	GO:0015935	Small ribosomal subunit	CC	3.1E-7	2.1E-6	***
	GO:0000049	tRNA binding	MF	1.5E-6	1.1E-5	***
	GO:0015934	Large ribosomal subunit	CC	1.8E-3	1.4E-2	***
	GO:0003917	DNA topoisomerase type I activity	MF	5.3E-3	4.3E-2	***

Fisher Exact Test was used with distinct statistically methods: False Discovery Rate control (FDR), Family Wise Error Rate (FWER) and the p-value without multiple testing corrections (single test p-value). Only the most specific GO terms overrepresented in each bacterial group using FDR with statistical significance (**p-value <0.01; ***p-value <0.001) are shown. Five bacterial groups were analyzed: Cyanobacteria, Gammaproteobacteria, Alphaproteobacteria; Flavobacteria; and non-marine Bacteroidetes.

Alphaproteobacteria GIs were enriched (six GO terms) in genes related to transposases, DNA transposition activity, and motility. Surprisingly, RNA-directed DNA-polymerase activity (a signature for the presence of retrovirus prophages) and ribosome assemblage, related to a high number of ribosomal proteins, were also specifically enriched in marine Alphaproteobacteria genomes. The two GO terms enriched in Gammaproteobacteria included genes associated to site-specific recombinases and ligase activity associated with DNA mobility and rearrangements.

Flavobacteria were specifically enriched in eight GO terms with genes associated to ATPase and GTPase activity and, interestingly, in processes related to DNA-directed RNA polymerases activity, transcription, and its regulation processes (Table 2). GIs of non-marine Bacteroidetes were enriched in seven GO terms, involved in translation processes and the structure of the ribosome (many ribosomal proteins associated with the large and small ribosomal subunits) and rRNA and tRNA binding.

Therefore, each bacterial class contains a different set of genes in their GIs, suggesting a different ecological strategy played by their GIs. This will be analyzed in the next section.

### Biologically relevant genes within marine bacterial GIs

**Figure 10:**
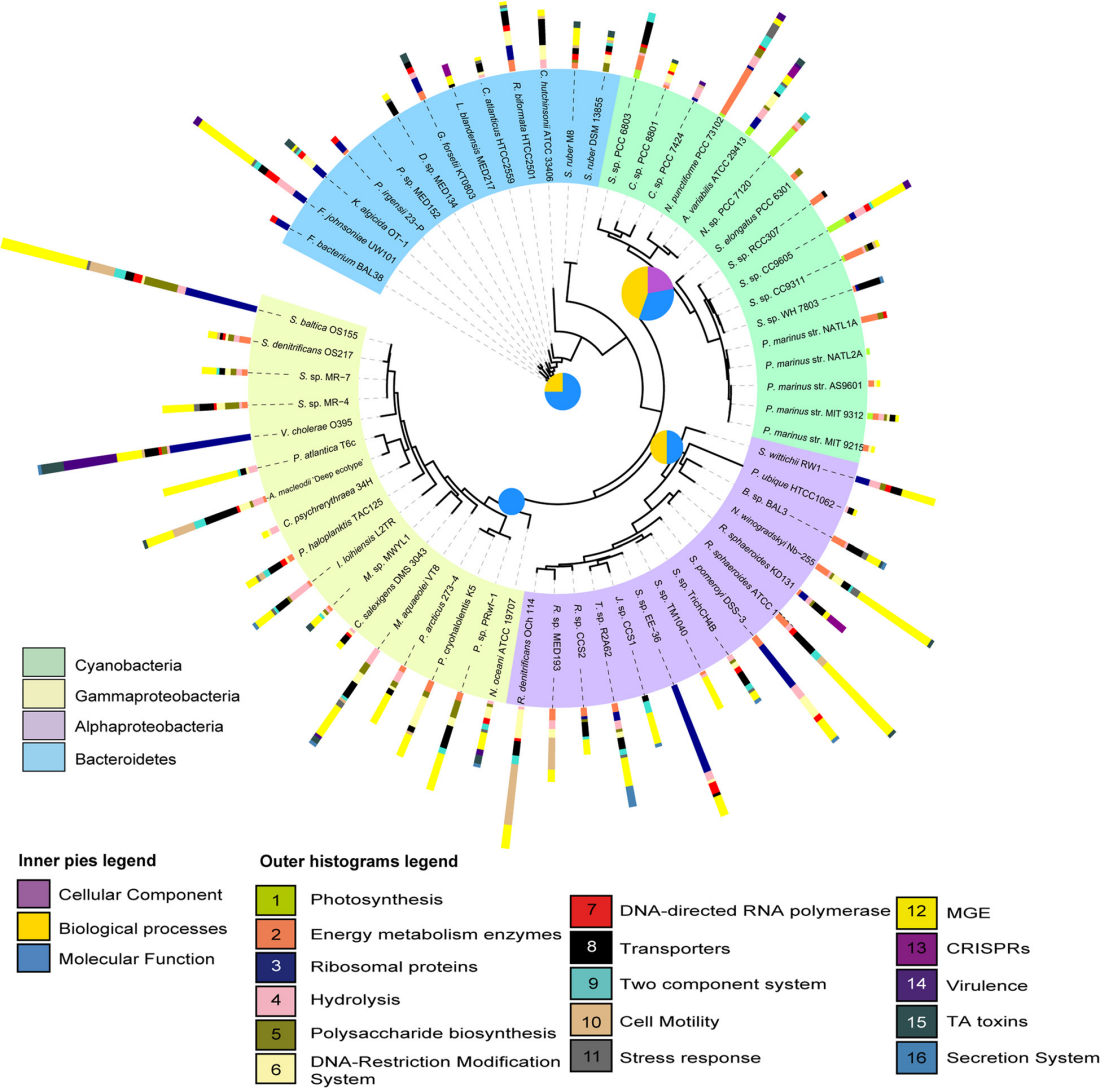
**Phylogenetic view of the 66 bacterial genomes with GIs.** The colored ring shows the four main phylogenetic groups analyzed; the histograms indicate the number of genes (in absolute number) within GIs associated with any of the 16 biological categories described. The inner pies show the functional category specifically overrepresented for Cyanobacteria, Gammaproteobacteria, Alphaproteobacteria and Flavobacteria wherein pie size is proportional to the number of GO functional categories enriched.

In order to analyze the ecological relevance of the genes found within GIs, we assigned them to 16 biological categories with the potential to increase bacterial fitness (see the complete list in Material and Methods). In Figure 10 shows a summary of the numbers of genes associated to each category in each genome analyzed (histograms in Figure 10). Some of these biological categories were widely distributed among the bacterial taxa, such as energy metabolism (number 2 in the histogram of Figure 10), ribosomal proteins (3), hydrolysis activity (4), DNA restriction modification systems (6), β-subunit of DNA-directed RNA polymerase (7), transporters (8), two component systems (9), stress response proteins (11) or MGE (12). Such categories were well distributed among the genomes but exhibited differences in their abundance within the GIs.

Transposases and integrases (category 12 in Figure 10) can modify the structure of the genome through the transfer of DNA sequences to new locations within or between genomes [59]. Recently, it has been reported that transposases are the most abundant and ubiquitous genes in nature [60]. We found a total of 675 genes related to transposases such as IS elements or transposons representing about 8.2% of the total database. These genes were overrepresented in Alphaproteobacteria within GIs (see significance test in Table 2) with almost a 30% of total transposases (194 genes) within this group (data not shown). Also, ribosomal proteins (category 3 in Figure 10) accounted for 3% of all genes in our dataset (253 out of 8152 genes) and were very abundant in Alphaproteobacteria and Gammaproteobacteria genomes (also in non-marine Bacteroidetes) with almost a 25% and 22% respectively (data not shown). Specifically, marine genomes with more than 10 gene copies were *Sulfitobacter* sp. EE-36 (32 copies) and *Ruegeria pomeroyi* DSS-3 (18 copies) in the Alphaproteobacteria, and *Shewanella baltica* OS155 (26 copies) and *Vibrio cholerae* O395 (29 copies) in the Gammaproteobacteria.

Virulence gene clusters (category 14 in Figure 10) were found in all main taxa except for Alphaproteobacteria with the highest number for *Vibrio cholerae* O395 with 19 copies corresponding to the well known TCP (Toxin-Coregulated Pilus) located in one of the pathogenic island organized as a prophage [61]. Finally, the presence of secretion system proteins (category 16 in Figure 10) was found in all taxa but marine Flavobacteria. Specifically, *Thalassiobium* sp. R2A62 exhibited seven copies of Type I secretion system proteins and at least 3 copies of type III secretion system were found in *Shewanella baltica* OS155. These virulence associated secretion system proteins have been found in other marine genomes. Particularly, type IV and type VI secretions system genes were recurrent for Alpha- and Gammaproteobacteria genomes respectively [29].

Our GI dataset also included genes involved in protection from bacteriophages such as DNA modification restriction systems (type I, II and III, category 6 in Figure 10). These systems are sequence-specific restriction enzymes, also called restriction endonucleases that have been known for a long time to act as a protection from foreign DNA, such as bacteriophages [62]. We detected 99 genes linked to restriction modification systems spread along all taxa but specially represented in: *Anabaena variabilis* ATCC 29413 with five copies, *Roseobacter denitrificans* OCh 114 (10 copies), *Ruegeria pomeroyi* DSS-3 (eight copies) and *Psychrobacter cryohalolentis* K5 (11 of them). Finally, three marine Bacteroidetes had five copies each (*Cytophaga hutchinsonii* ATCC 33406, *Kordia algicida* OT-1 and *Robiginitalea biformata* HTCC2501) (Figure 10). Interestingly, most of the restriction-modification system genes in our GI dataset were of the type I (the most complex) with at least 36 genes, but we also found some representatives of type II and III (data not shown).

Another mechanism proposed to confer resistance from phage and possibly from other mobile elements is the presence of CRISPR systems with their associated *cas* genes [63-65]. These genetics elements have been identified in approximately 40% and 90% of Bacteria and Archaea genomes, respectively [66]. Recent research has shown that CRISPR systems could be primarily transferred by horizontal gene transfer and can be found overrepresented within GIs [13]. Although we did not find many of them (category 13 in Figure 10) associated to our marine prokaryotic GI dataset, we found *cas* genes in *Anabaena variabilis* (four copies), in *Rhodobacter sphaeroides* KD131 (seven copies) and four genes in *Leeuwenhoekiella blandensis* MED217. Some of these CRISPR systems were already in the CRISPRdb ( http://crispr.u-psud.fr/crispr/) in the chromosome of these genomes although they were not associated with GIs and no CRISPR had been previously identified in *Leeuwenhoekiella blandensis* MED217.

Other categories were restricted to a few genomes and/or phylogenetic groups. This was the case of photosynthetic genes (category 1 in Figure 10) within cyanobacterial taxa. Photosystem I subunits and photosynthesis antenna proteins as well as electron transporter systems (ATP synthases) or ferredoxins were detected in Cyanobacteria GIs. Photosynthesis genes within GIs have been well described in *Prochlorococcus* and *Synechococcus* strains [5,6,9] but we also found them in *Synechocystis* sp. PCC 6803 with three phycobilisome linker proteins in the 14.6 kb GI, as well as in *Anabaena variabilis* ATCC 29413 with two photosystem I subunit proteins and one ferredoxin, and in *Nostoc* sp. PCC7120 with eight ATP synthase subunits, two phycobilisome linker proteins and another two allophycocyanin alpha/beta subunits. These photosynthetic genes are linked to relevant physiological characteristics of these photoautotrophic bacteria. The acquisition of these genes by GIs may provide specific light niche adaptations to specific strains [6], underlying the need for analyzing their GIs to fully understand the ecology of Cyanobacteria.

In addition, we detected cell motility (flagellum) genes (category 10 in Figure 10) constrained basically to *Roseobacter denitrificans* OCh 114 and *Roseobacter* sp. MED193 with 20 and 11 copies, respectively, and *Shewanella baltica* OS155 with eight. Cell motility by flagella is an important physiological trait that allows bacteria to move towards favorable environmental conditions, form biofilms and/or acquire nutrients. Genes to reconstruct the flagellum structure can include more than 50 but only 24 are considered to be the core set and are present in most flagellated bacterial taxa [67]. Some of theses genes can be acquired through HGT events as described in *Photobacterium profundum* SS9 [23]. In this organism a cluster of genes involved in the lateral flagellar synthesis was present in the GI and absent in a closely related strain, suggesting that it could have been horizontally transferred. Accordingly, 43 genes within two *Roseobacter* genomes and two Gammaproteobacteria might have followed the same fate. Genes related to the flagellar basal rod, body, ring, and hook or flagellin proteins were repeatedly found in these GIs. In *Roseobacter denitrificans* OCh 114, 14 of these flagellar genes were concentrated in two GIs of 14.1 kb and 16 kb with 12 and 8 genes respectively. These GIs were flanked by transposase, integrase or phage- integrase genes, while in *Roseobacter* sp. MED193 these genes were in a single GI 12 kb long, also flanked by phage-integrase genes. In addition, *Shewanella baltica* OS155 had eight flagellar related genes in the GI of 11.5 kb and interestingly, a chemotaxis protein gene was located within the same GI. Moreover, one of the GIs (18.7 kb) of *Alteromonas macleodii* "deep ecotype" displayed eight flagellar protein gene in GI8 (20 kb) previously reported in reference [25]. We investigated whether these flagellar protein genes present in our GIs dataset were also present in the genome but we found different genes for the flagellum structure in the chromosome (data not shown). Finally, conjugative transposon genes (category 12 in Figure 10) were found only in a few taxa. Two Alphaproteobacteria, *Jannaschia* sp. CCS1 and *Sphingomonas wittichii* RW1 displayed six and seven genes related to these conjugative transposons, and the Bacteroidetes *Flavobacterium johnsoniae* UW101 had four copies.

It is highly probable that many genes within GIs are positively selected due to the potential benefits of these genes for the life-style of their host. It has been shown that GIs of *Prochlorococcus* strains displayed differential expression under light and nutrient stress conditions [9]. Also, *Prochlorococcus* GIs contain genes related to the attachment of virions to the host cell surface and those GIs have an important role in the viruses-host coexistence [11]. Moreover, one of the GIs with heavy metal resistance genes detected in *Alteromonas macleodii* "deep ecotype" conferred to this strain higher resistance to mercury and zinc concentrations [25]. In a different context, in the Actinobacteria *Salinispora arenicola,* orthologs within GIs showed evidence of positive selection compared with the non-island genes (7.6% vs. 1.6%) [20]. Also, the hyperhalophilic bacterium *Salinibacter ruber* strains M8 and M31 displayed 40 strains-specific genes present in their GIs with a ratio of substitution rates at non-synonymous and synonymous sites >1 (dN/dS >1) in which 25 of them were HPs [37]. Consequently, there is some evidence for the adaptive significance of GI genes among environmental bacteria, but the extent and effect on diversification mechanisms in marine bacterial taxa is still unclear.

## Conclusions

GIs were present in most of the marine bacterial genomes analyzed. Our results indicated that both horizontal gene transfer by phages, plasmids and MGE and HR play an important role for the mobility of clusters of genes between taxa and within closely related genomes, thus modulating the flexible pool of the genome. Our findings provide insights into the possible role of GIs to increase bacterial fitness under changing environmental conditions by providing, not only novel foreign genes, but also modulating their transcription, regulation, and/or transduction. The potential role that GIs have in rearranging the structure, and increasing the diversity, of marine bacterial genomes is emphasized by the results presented here. We observed that some GIs were intimately associated with the physiology and ecology of the microorganisms but we also found some relevant conserved genes in theory linked to the core genome. These results would reinforce the need to establish a pangenome concept for marine bacterial species wherein GIs would be crucial to fully understand the ecology and evolution of marine bacteria in the ocean. Exploring the mechanisms maintaining and selecting GIs is the next logical step to gain insights into the evolutionary processes shaping marine bacterial genomes.

## Methods

### GI prediction and database construction

Seventy prokaryotic genomes were analyzed in this study. GIs of 53 genomes were obtained at the time of our analyses (February 2010) directly from IslandViewer database ( http://www.pathogenomics.sfu.ca/islandviewer). IslandViewer is a web-based interface that integrates several methods for identification and visualization of GIs: IslandPick, IslandPath-DIMOB and SIGI-HMM [34]. IslandPick [33] is a comparative GIs prediction method that requires phylogenetically related genomes to be available for the comparison. SIGI-HMM measures codon usage [34] and IslandPath [35] the abnormal sequence composition or the presence of genes related to mobile elements to identify possible GIs. For a recent review of Bioinformatics approaches to detect GIs see [32]. The remaining 17 genomes (eight Alphaproteobacteria and nine marine Bacteroidetes) were downloaded from the National Center for Biotechnology Information (NCBI; http://www.ncbi.nlm.nih.gov) and the J. Craig Venter Institute ( http://www.jcvi.org/). Fourteen of these 17 genomes were not closed. The genomes that were not available in IslandViewer were uploaded and the GIs predicted by IslandPick by selecting closely related genomes (at least three genomes when possible) plus one reference distant genome. IslandPick GIs prediction detects also the GIs that overlap with the other two GIs predictors, IslandPath-DIMOB and SIGI-HMM [34] and this information was also integrated in our database. The 438 GIs detected are shown in Additional file 2 including predictor methods used to detect them and some features such as the presence of MGE like plasmids, transposases, integrons, conjugative transposons or phages. Most of the GIs were detected by at least two of the three methods integrated in IslandViewer. In addition, manual refining of these GIs was carried out in Artemis and Integrated Microbial Genomes (IMG) genome browser, by including the presence of tRNA within or flanking the GIs detected. GIs of the 70 bacterial genomes were exported in csv format and all proteins in a fasta file. The genome selection criteria used were: (i) presence of marine genomes with pre-calculated GIs within IslandViewer, (ii) availability when possible of at least three closely related genomes, and (ii) the widest possible taxonomic representation of ecologically relevant marine bacteria.

### Phylogenetic analyses

Maximum likelihood (ML) phylogenetic analyses of selected genomes were carried out on full-length 16 S rRNA gene sequences, and the elongation factor Tu (EF-Tu) gene and the ß subunit of the RNA polymerase (RpoB) on amino acid sequences. The sequences were aligned using MAFFT [v.6.857] with the algorithm E-INS-I [68]. An additional more stringent alignment was constructed by removing ambiguously aligned sites using Gblocks [69] as well as visual examination. Phylogenies were constructed using ML as implemented in RAxML [v.7.2.8] [70] with GTR nucleotide substitution model for the 16S rRNA sequences and the BLOSUM62 amino acid substitution matrix for the EF-Tu and RpoB sequences. The trees generated were visualized and edited in Interactive Tree Of Life [71].

### GIs analyses in previous studies (control) vs. this study

We selected eight genomes found in the public sequence databases as control genomes, in which the GIs had been described, to test the accuracy of our approach for GI prediction in marine genomes even when 3 closely related genomes were not always available for comparison. We calculated several parameters such as % overlap, precision, recall (sensitivity) as detailed in Langille et al. [33] shown in Table 1. The GIs of control genomes were verified by different approaches (see Table 1). Comparison of the control and automatically predicted GIs was carried out using the genomic display software CIRCOS [72].

### Functional annotation of GIs

Gene sequences found in the predicted GIs were extracted and stored in a flat file-type database. The potential protein domains were analyzed using the package HMMER 3.0 [73] against the PFAM 24.0 database [74]. Clusters of orthologous groups of proteins (COGs) for characterization of the proteins [75] were carried out using the rpsblast program bundled in the NCBI BLAST package with an E-value of 1E-5 as threshold [76]. In addition, Blast2GO (B2G) was used to run the functional annotation of the extracted sequences with an E-value of 1E-20 and cut-off identity in their amino acid sequences of 55% [77,78]. Gene ontologies [79] and EC number from KEGG pathways [80] were retrieved to identify the main biological processes, molecular functions, and cellular components present in the GIs.

### Statistical and GIs comparison analyses

Both total genome peptides and peptides within GIs were classified by BLASTP hits to the NCBI's Cluster of Orthologous Genes (COG) as described in [81] with cutoffs of E-value ≤1E-5, identity ≥ 30 and coverage ≥ 50%. HPs were considered as those with no hits to the COG database. To estimate protein functions that were overrepresented in different taxonomic groups we used the Gossip package [82] implemented in the Bioinformatics annotation tool Blast2GO [77]. This package uses the Fisher´s Exact Test with multiple testing corrections and, since multiple categories are examined simultaneously, the Benjamini and Hochberg False Discovery Rate correction (FDR) for multiple testing was determined for all functional category analyses. We considered p-values smaller than 0.01 to be significant (p <0.01). Fisher´s Exact Test (FT) was used to explore putative significant differences at three comparative levels: (i) the functional gene annotation of the combined GIs of each control genome and their corresponding detected automatically GIs, (ii) the combined GIs dataset for the eight control genomes and their corresponding automatically detected GIs and (iii) the comparative gene enrichment analyses among the bacterial taxa of Cyanobacteria, Gammaproteobacteria, Alphaproteobacteria and marine and non-marine Bacteroidetes to detect functional categories enriched within each taxa.

In addition, we assigned genes within GIs to 16 biological categories defined by us based on the most representative GO terms with ecologically relevance and related to: photosynthesis (1) (photosystem, antenna proteins and electron transport system), energy metabolism enzymes (2), ribosomal proteins (3), hydrolysis (4), polysaccharide biosynthesis (5), DNA restriction modification system (type I, II & III) (6), DNA-directed RNA polymerases (7), transporters (ABC and multridrug/metal resistence) (8), two component system (9), cell motility (flagellum and chemotaxis) (10), stress response (heat shock or chaperone proteins) (11), MGE (conjugative transposon, integrases, phage integrases, transposon Tn21/Tn7) (12), CRISPRs (13), virulence (14), TA toxins (plasmid killer system) (15), and secretion systems proteins (type I, II and III) (16). A matrix was built showing the number of genes within GI assigned to each of the 16 biological categories (Additional file 9). GO terms enrichment analyses and the matrix of ecological gene categories, were implemented in the iTOL software for visualization ( http://itol.embl.de/).

## Abbreviations

GIs: Genomic Islands; HGT: Horizontal Gene Transfer; HR: Homologous Recombination; CRISPR: Clustered Regularly Interspaced Palindromic Repeat; COG: Cluster of Orthologous Groups; GO: Gene Ontology; MGE: Mobile Genetic Element; HP: Hypothetical Protein; CC: Cellular Component; BP: Biological Process; MF: Molecular Function; RpoB: β-subunit of DNA-directed RNA polymerase; EF-Tu: elongation factor Tu; NCBI: National Center for Biotechnology Information; IMG: Integrated Microbial Genomes; ML: Maximum Likelihood.

## Competing interests

The authors have declared no competing interests exist.

## Authors´ contributions

SGA conceived and designed the analyses. BF-G, AF-G, JMG and SGA performed the analyses and analyzed the data. All authors helped in interpreting the data. SGA wrote the paper with significant contributions from all authors with special mention to CPA. All authors read and approved the final manuscript.

## Supplementary Material

Additional file 1 Total number of genes and genes whose function could be assigned in the set of 8 control genomes previously published and genomes from this study. Click here for file

Additional file 2 Table listing the 438 GIs detected in our selected 70 marine bacterial genomes including the method to predict them along with some features of the GIs. Click here for file

Additional file 3 List of the 70 bacterial genomes used in this study indicating the number of GIs detected, the total GI size and the percentage of the bacterial genome represented by GIs. Click here for file

Additional file 4 Relation between total GI size and genome size in four main phylogenetic groups. Click here for file

Additional file 5 Phylogeny of the 16S rRNA gene of 20 Bacteroidetes genomes that contain the HR1-GI. Click here for file

Additional file 6 Phylogenetic comparison of the 16S rRNA, EF-Tu and RpoB genes in 20 Bacteroidetes genomes. Click here for file

Additional file 7 **Phylogenetic reconstruction based on the EF-Tu gene of 19 * Shewanella* strains that contain HR-GI in their genomes.** Click here for file

Additional file 8 Table listing bacterial taxa specific gene enrichment analyses. Click here for file

Additional file 9 Matrix with the number of genes within GIs assigned to each of the 16 biological categories for all marine bacterial genome analyzed. Click here for file
