# Supplementary material for: Patterns and architecture of genomic islands in marine bacteria
Source: BMC Genomics. 2012 Jul 29;13:347. doi: 10.1186/1471-2164-13-347 (PMC3478194; doi:10.1186/1471-2164-13-347)
Supplement: Additional file 1 — Total number of genes and genes whose function could be assigned in the set of 8 control genomes previously published and genomes from this study. [file 1471-2164-13-347-S1.docx]

**Additional Data File 1**. Total number of genes and genes where function could be assigned for available published GIs and the GIs detected in this study for 8 control genomes.

| **Control genome** | **Published GIs** | | | | **This study GIs** | | | **Fisher´s Test** | |
| --- | --- | --- | --- | --- | --- | --- | --- | --- | --- |
|  | **Total genes** | **Genes with known function^a^** | | | **Total genes** | **Genes with known function^a^** | | **Significant difference** | |
| *Prochlorococcus marinus str. MIT9312* | 239 | | 82 | 44 | | | 16 | | NO |
| *Synechococcus sp. WH7803* | 423 | | 154 | 75 | | | 26 | | NO |
| *Synechococcus sp. CC9311* | 705 | | 225 | 128 | | | 51 | | NO |
| *Synechococcus sp. CC9605* | 565 | | 159 | 323 | | | 79 | | NO |
| *Synechococcus sp. RCC307* | 448 | | 138 | 56 | | | 28 | | NO |
| *Alteromonas macleodii*  “Deep ecotype” | 467 | | 190 | 276 | | | 116 | | NO |
| *Salinibacter ruber* M8 | 282 | | 51 | 171 | | | 33 | | NO |
| *Salinibacter ruber* DSM13855 | 158 | | 66 | 101 | | | 48 | | NO |

Significance (p-value <0.01) of Fisher´s exact test was used to detect significant differences between them.

**^a^** The difference with total genes is accounted for by hypothetical proteins.
