# Supplementary material for: Patterns and architecture of genomic islands in marine bacteria
Source: BMC Genomics. 2012 Jul 29;13:347. doi: 10.1186/1471-2164-13-347 (PMC3478194; doi:10.1186/1471-2164-13-347)
Supplement: Additional file 3 — List of the 70 bacterial genomes used in this study indicating the number of GIs detected, the total GI size and the percentage of the bacterial genome represented by GIs. [file 1471-2164-13-347-S3.docx]

Table 3SM. List of the 70 bacterial genomes used for our analyses with the number of genomic islands detected, total GI size (in kb) and the percentage of the bacterial genome represented by GIs.

| **Bacterial genomes** | Genome **Size (Mb)** | **# of GIs (≥9.5 kb)^a^** | **GIs Size**  **(kb)** | **Ratio***  **(%)** |
| --- | --- | --- | --- | --- |
| **Cyanobacteria (16)** |  |  |  |  |
| *Anabaena variabilis* ATCC 29413 | 6.36 | 9 | 177.61 | 2.79 |
| *Cyanothece* sp. PCC 7424 | 5.94 | 4 | 110.28 | 1.86 |
| *Cyanothece* sp. PCC 8801 | 4.67 | 2 | 24.65 | 0.53 |
| *Nostoc punctiforme* PCC 73102 | 8.23 | 12 | 243.35 | 2.96 |
| *Nostoc* sp. PCC 7120 | 6.43 | 3 | 49.01 | 0.76 |
| *Prochlorococcus marinus* str. AS9601 | 1.67 | 1 | 9.99 | 0.6 |
| *Prochlorococcus marinus* str. MIT 9215 | 1.73 | 1 | 10.47 | 0.61 |
| *Prochlorococcus marinus* str. MIT 9312 | 1.7 | 3 | 36.93 | 2.17 |
| *Prochlorococcus marinus* str. NATL2A | 1.84 | 1 | 18.18 | 0.99 |
| *Prochlorococcus marinus* str. NATL1A | 1.86 | 3 | 36.63 | 1.97 |
| *Synechococcus elongatus* PCC 6301 | 2.69 | 1 | 10.14 | 0.38 |
| *Synechococcus* sp. CC9311 | 2.6 | 2 | 58.37 | 2.24 |
| *Synechococcus* sp. CC9605 | 2.51 | 11 | 175.45 | 6.99 |
| *Synechococcus* sp. RCC307 | 2.22 | 1 | 14.87 | 0.67 |
| *Synechococcus* sp. WH 7803 | 2.36 | 2 | 21.87 | 0.93 |
| *Synechocystis* sp. PCC 6803 | 3.57 | 5 | 110.91 | 3.11 |
| **Gammaproteobacteria (17)** |  |  |  |  |
| *Alteronomas macleodii* ‘deep ecotype’ | 4.2 | 10 | 260.47 | 6.20 |
| *Chromohalobacter salexigens* DMS 3043 | 3.69 | 4 | 57.97 | 1.57 |
| *Colwellia pshychrerythraea* 34H | 5.37 | 3 | 72.81 | 1.36 |
| *Idiomarina loihiensis* L2TR | 3.83 | 5 | 90.13 | 2.35 |
| *Marinobacter aquaeoloi* VT8 | 4.32 | 17 | 258.13 | 5.98 |
| *Marinomonas* sp. MWYL1 | 5.1 | 5 | 127.88 | 2.51 |
| *Nitrosococcus oceani* ATCC 19707 | 3.48 | 7 | 178.25 | 5.12 |
| *Pseudoalteronomas atlantica* T6c | 3.5 | 3 | 98.50 | 2.81 |
| *Pseudoalteromonas haloplanktis* TAC125** | 3.83 | 5 | 101.68 | 2.65 |
| *Psychrobacter arcticus* 273-4 | 2.65 | 7 | 132.28 | 4.99 |
| *Psychrobacter cryohalolentis* K5 | 3.06 | 5 | 193.26 | 6.32 |
| *Psychrobacter* sp. PRwf-1 | 2.98 | 10 | 204.71 | 6.87 |
| *Shewanella baltica* OS155 | 5.12 | 22 | 323.04 | 6.31 |
| *Shewanella baltica* sp. MR-4 | 4.7 | 9 | 177.94 | 3.79 |
| *Shewanella baltica* sp. MR-7 | 4.07 | 9 | 162.43 | 3.99 |
| *Shewanella denitrificans* OS217 | 4.54 | 7 | 110.83 | 2.44 |
| *Vibrio cholerae* O395** | 4.12 | 10 | 265.41 | 6.44 |
| **Alphaproteobacteria (16)** |  |  |  |  |
| *Brevundimonas* sp. BAL3 | 3.63 | 6 | 114.08 | 3.14 |
| Candidatus *Pelagibacter ubique* HTCC1062 | 1.3 | 1 | 13.36 | 1.03 |
| *Jannaschia* sp. CCS1 | 4.31 | 7 | 147.01 | 3.41 |
| *Nitrobacter winogradskyi* Nb-255 | 3.4 | 13 | 234.24 | 6.89 |
| *Pelagibacter* sp. HTCC7211 | 1.4 | 0 | 0 | 0 |
| *Rhodobacter sphaeroides* ATCC17025 | 3.21 | 14 | 389.03 | 12.12 |
| *Rhodobacter sphaeroides* KD131** | 4.34 | 9 | 130.48 | 3.01 |
| *Roseobacter denitrificans* OCh 114 | 4.1 | 9 | 221.78 | 5.41 |
| *Roseobacter* sp. CCS2 | 3.49 | 4 | 63.83 | 1.83 |
| *Roseobacter* sp. MED193 | 4.65 | 6 | 83.90 | 1.8 |
| *Ruegeria pomeroyi* DSS-3 | 4.1 | 7 | 194.32 | 4.74 |
| *Silicibacter* sp. TM1040 | 3.2 | 1 | 29.64 | 0.93 |
| *Silicibacter* sp. TrichCH4B | 4.67 | 6 | 109.43 | 2.34 |
| *Sphingomonas wittichii* RW1 | 5.38 | 8 | 181.36 | 3.37 |
| *Sulfitobacte*r sp. EE-36 | 3.54 | 4 | 114.15 | 3.22 |
| *Thalassiobium* sp. R2A62 | 3.48 | 10 | 185.42 | 5.33 |
| **Bacteroidetes (21)**  **(14 Flavobacteria + 7 Non Flavobacteria)** |  |  |  |  |
| *Bacteroides fragilis* NCTC 9343 | 5.2 | 13 | 253.27 | 4.87 |
| *Bacteroides fracilis* YCH46 | 5.3 | 15 | 274.44 | 5.18 |
| *Bacteroides thetaiothaomicron* VPI-5482 | 6.2 | 22 | 340.91 | 5.50 |
| *Bacteroides vulgatus* ATCC 8482 | 5.1 | 22 | 436.62 | 8.56 |
| *Porphyromonas gingivalis* ATCC 33277 | 2.3 | 7 | 124.94 | 5.43 |
| *Salinibacter ruber* DSM 13855 | 3.5 | 6 | 154.4 | 4.41 |
| *Salinibacter ruber* M8 | 3.6 | 7 | 123.54 | 3.43 |
| *Croceibacter atlanticus* HTCC2559 | 2.9 | 1 | 41.81 | 1.44 |
| *Cytophaga huchinsonii* ATCC33406 | 4.4 | 4 | 136.96 | 3.11 |
| *Dokdonia* sp. MED134 | 3.3 | 2 | 38.98 | 1.18 |
| *Flavobacteria bacterium* BBFL7 | 3.1 | 0 | 0 | 0.00 |
| *Flavobacteriales bacterium* ALC-1 | 3.8 | 0 | 0 | 0.00 |
| *Flavobacterium bacterium* BAL38 | 2.8 | 3 | 49.464 | 1.77 |
| *Flavobacterium johnsoniae* UW101 | 6.1 | 9 | 247.25 | 4.05 |
| *Flavobacterium psychrophilum* JIP02/86 | 2.8 | 0 | 0 | 0.00 |
| *Gramella forsetii* KT0803 | 3.8 | 5 | 105.29 | 2.77 |
| *Kordia algicida* OT-1 | 5 | 7 | 173.13 | 3.46 |
| *Leeuwenhoekiella blandensis* MED217 | 4.2 | 3 | 54.80 | 1.30 |
| *Polaribacter irgensii* 23-P | 2.7 | 1 | 14.90 | 0.55 |
| *Polaribacter* sp. MED152 | 2.9 | 1 | 12.51 | 0.43 |
| *Robiginitalea biformata* HTCC2501 | 3.5 | 6 | 157.84 | 4.51 |

* RATIO: Genomic islands size (kb) x100/Genome size (kb).

** Bacterial genomes with two chromosomes in which we have integrated all genomic islands found in both chromosomes.

^a^ We shown only those GIs **≥** 9.5 kb.
