# Supplementary figures and images for: Patterns and architecture of genomic islands in marine bacteria
Source: BMC Genomics. 2012 Jul 29;13:347. doi: 10.1186/1471-2164-13-347 (PMC3478194; doi:10.1186/1471-2164-13-347)

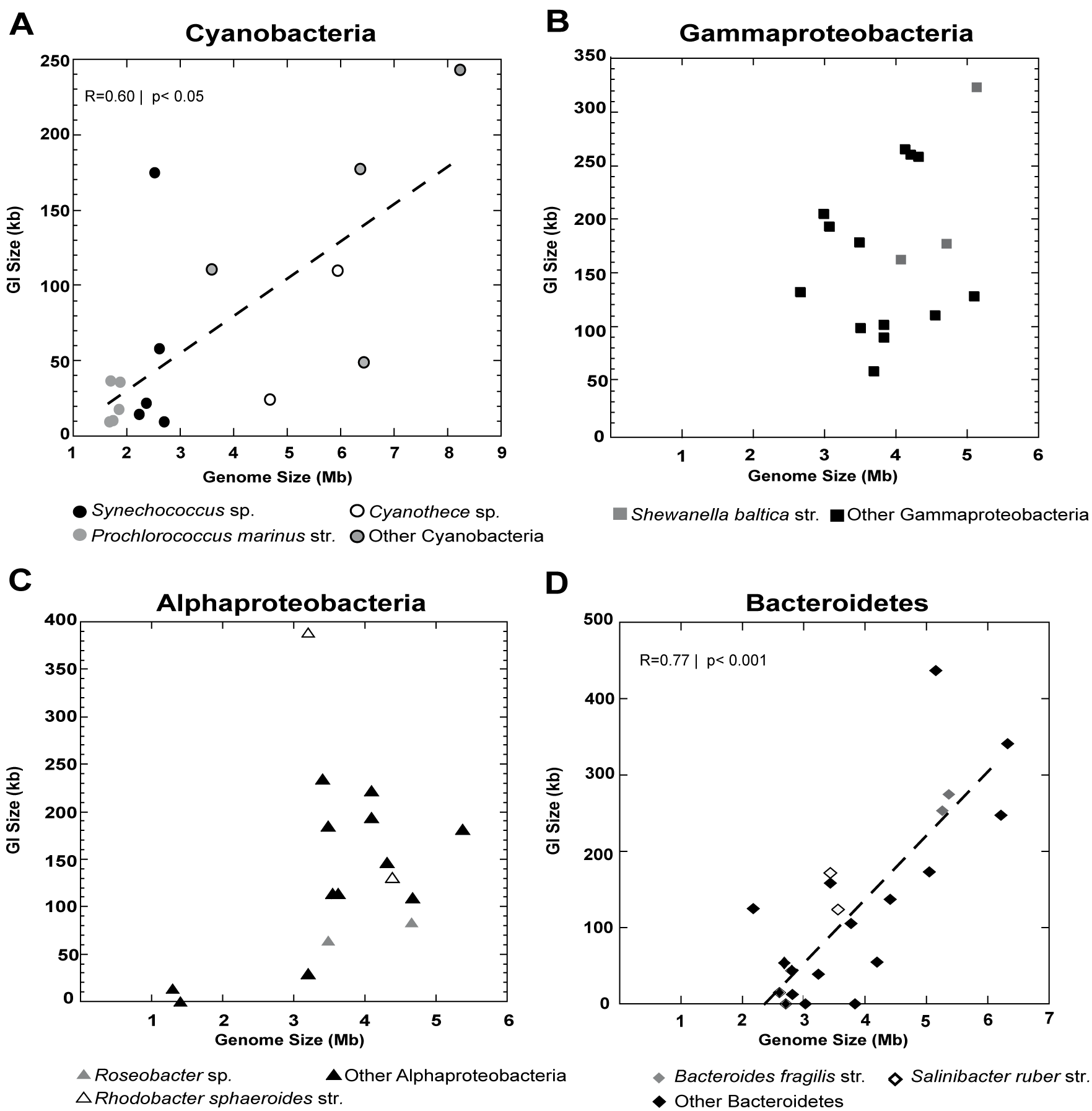

Supplement: Additional file 4 — Relation between total GI size and genome size in four main phylogenetic groups. [file 1471-2164-13-347-S4.tiff]

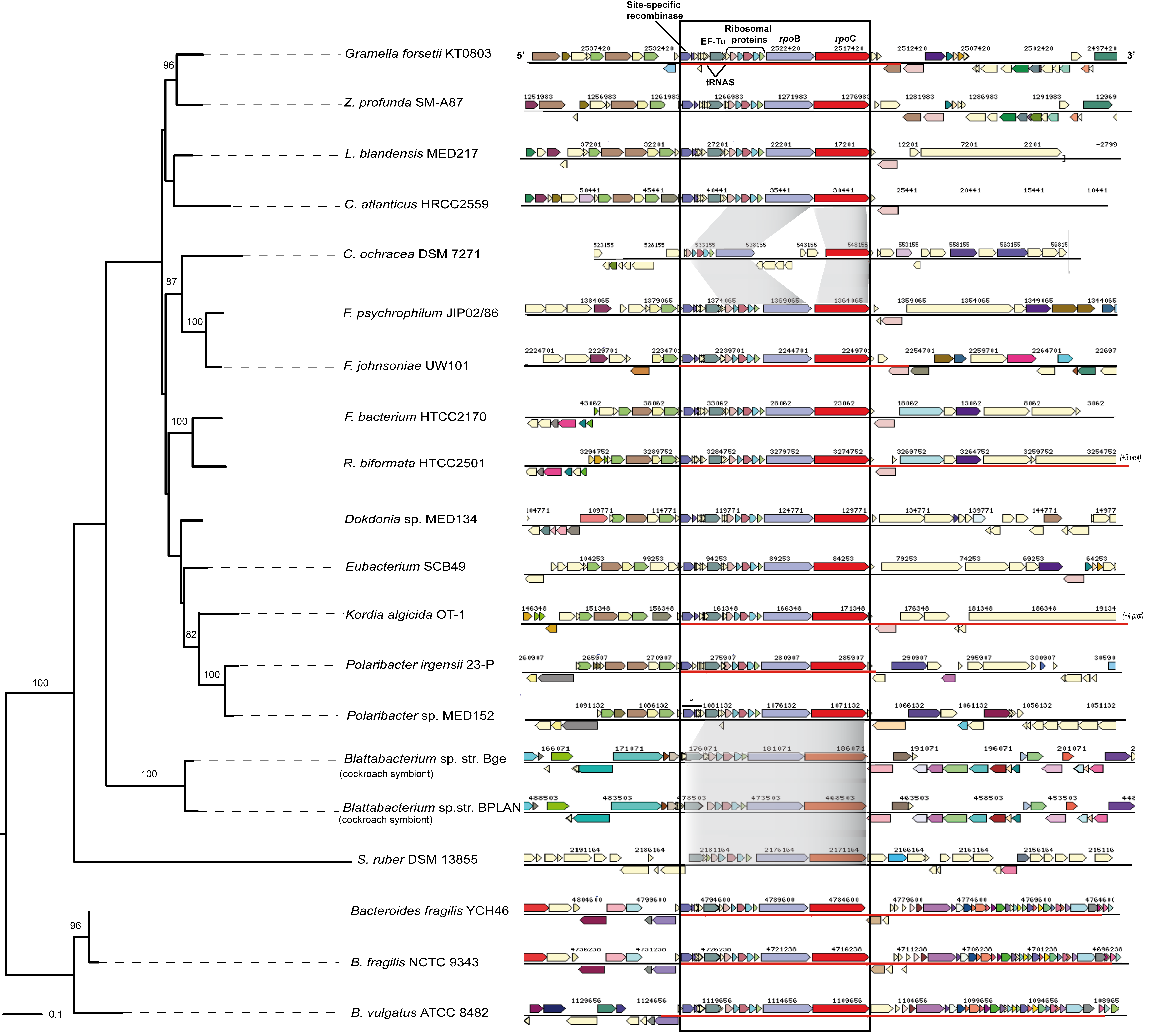

Supplement: Additional file 5 — Phylogeny of the 16S rRNA gene of 20 Bacteroidetes genomes that contain the HR1-GI. [file 1471-2164-13-347-S5.tiff]

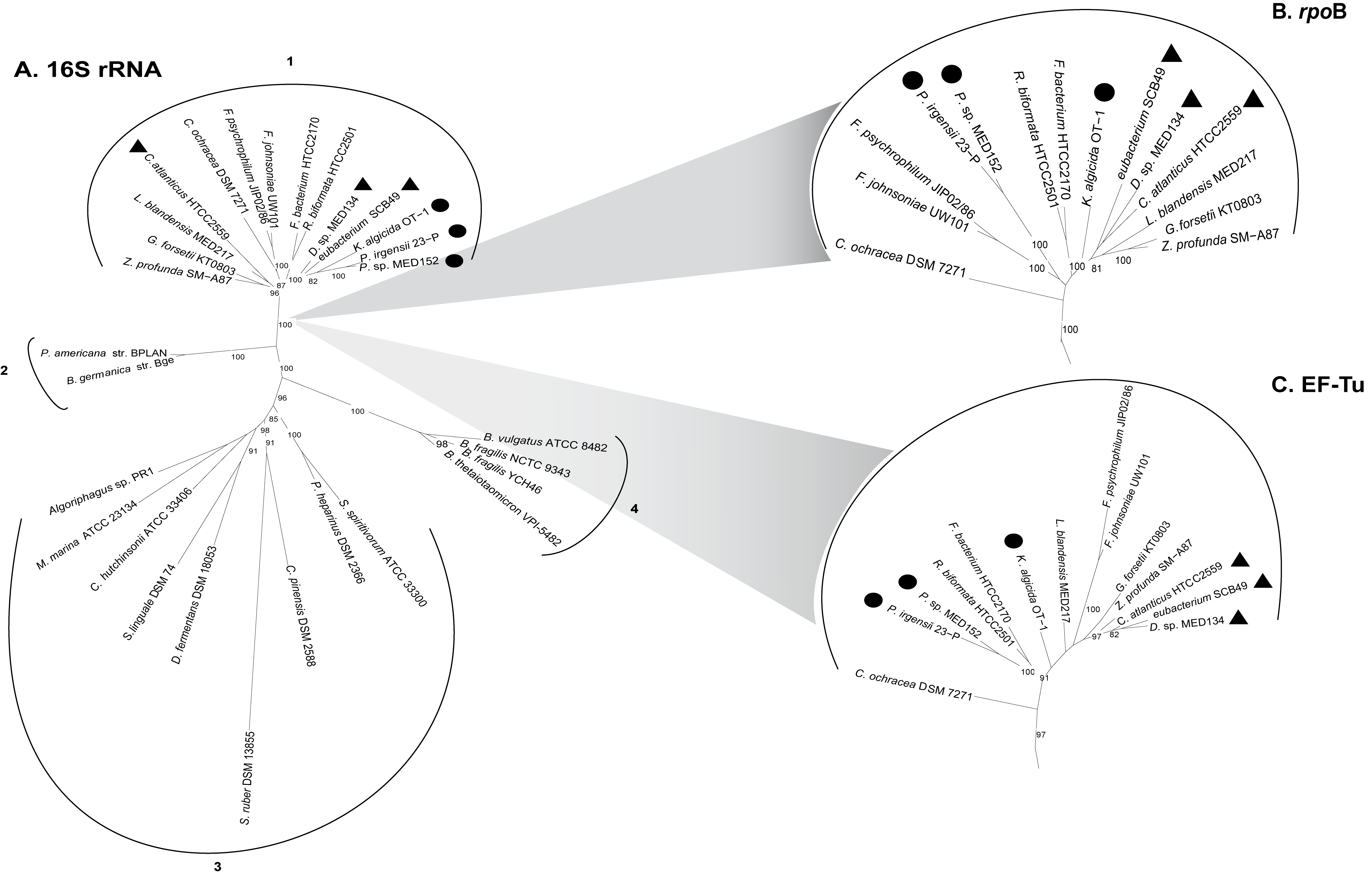

Supplement: Additional file 6 — Phylogenetic comparison of the 16S rRNA, EF-Tu and RpoB genes in 20 Bacteroidetes genomes. [file 1471-2164-13-347-S6.tiff]

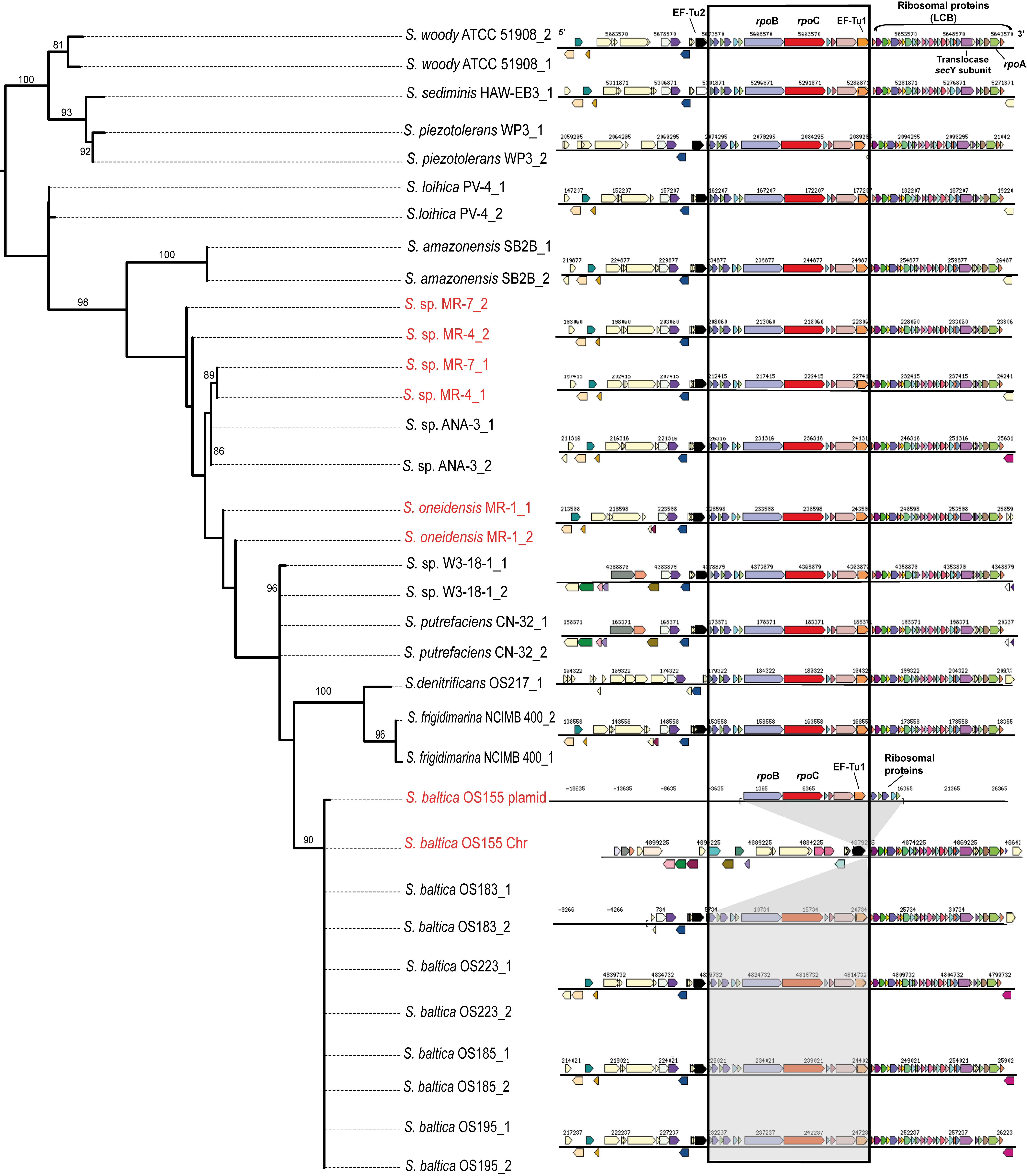

Supplement: Additional file 7 — Phylogenetic reconstruction based on the EF-Tu gene of 19 Shewanella strains that contain HR-GI in their genomes. [file 1471-2164-13-347-S7.tiff]
