# Supplementary material for: Patterns and architecture of genomic islands in marine bacteria
Source: BMC Genomics. 2012 Jul 29;13:347. doi: 10.1186/1471-2164-13-347 (PMC3478194; doi:10.1186/1471-2164-13-347)
Supplement: Additional file 8 — Table listing bacterial taxa specific gene enrichment analyses. [file 1471-2164-13-347-S8.docx]

**Additional Data File 8**. Table listing bacterial taxa specific gene enrichment analyses.

Fisher´s exact Test (FT) was used to detect bacterial taxa enriched genes within GIs by comparison between different combinations of bacterial groups. We referred as Reference Set (Ref) all genes within GIs for all phylogenetic groups except with the

taxa to compare with (Test Set).

*The five bacterial group tested are labeled as follow: A=Alphaproteobacteria; C=Flavobacteria; D= Non marine Bacteroidets; E= Cyanobacteria, G=Gammaproteobaacteria.

** Positive results of the Fisher´s exact Test. These categories with statistical significance (p-value <0.01) are listed in Table 2.

| **Bacterial Groups**  **Comparison*** | **# Genes in Reference Set** | **# Genes in**  **Test Set** | **# Annotated Genes (Ref/Test)** | **Fisher´s Significance**  **Test**** |
| --- | --- | --- | --- | --- |
| All-A (Ref) vs. A (Test) | 6211 | 2117 | 2523/837 | YES |
| All-C (Ref) vs. C (Test) | 7246 | 1082 | 3026/334 | YES |
| All-D (Ref) vs. D (Test) | 6863 | 1465 | 2894/466 | YES |
| All-E (Ref) vs. E (Test) | 7166 | 986 | 2875/404 | YES |
| All-G (Ref) vs. G (Test) | 5826 | 2502 | 2122/1238 | YES |
