# Supplementary material for: Patterns and architecture of genomic islands in marine bacteria
Source: BMC Genomics. 2012 Jul 29;13:347. doi: 10.1186/1471-2164-13-347 (PMC3478194; doi:10.1186/1471-2164-13-347)
Supplement: Additional file 9 — Matrix with the number of genes within GIs assigned to each of the 16 biological categories for all marine bacterial genome analyzed. [file 1471-2164-13-347-S9.docx]

**Additional Data File 9**. Matrix with the number of genes within GIs assigned to each of the 16 biological categories for all marine bacterial genomes analyzed.

| **Bacterial strain^a^** | **Photosynthesis^b^** | **Energy**  **Metabolism Enzymes** | **Ribosomal**  **Proteins** | **Hydrolysis** | **Polysacch.**  **Biosynthesis** | **DNA-Restrict.**  **Modif. System** | **DNA-directed RNA pol.** | **Transport** | **Two Comp.**  **System** | **Cell Motility** | **Stress**  **Response** | **MGE** | **CRISPR** | **Virulence** | **TA-**  **toxins** | **Secretion System** |
| --- | --- | --- | --- | --- | --- | --- | --- | --- | --- | --- | --- | --- | --- | --- | --- | --- |
| **ATCC 29413** | 3 | 0 | 4 | 4 | 1 | 5 | 0 | 1 | 3 | 0 | 1 | 3 | 4 | 1 | 2 | 0 |
| **PCC 7424** | 0 | 0 | 1 | 4 | 0 | 0 | 0 | 0 | 0 | 1 | 0 | 0 | 0 | 1 | 0 | 0 |
| **PCC 8801** | 0 | 1 | 0 | 1 | 0 | 3 | 1 | 0 | 0 | 0 | 0 | 3 | 0 | 0 | 1 | 0 |
| **PCC 73102** | 1 | 18 | 1 | 4 | 3 | 0 | 1 | 0 | 4 | 0 | 5 | 2 | 0 | 2 | 0 | 0 |
| **PCC 7120** | 12 | 1 | 0 | 2 | 2 | 0 | 0 | 1 | 2 | 0 | 0 | 0 | 0 | 0 | 0 | 0 |
| **AS9601** | 0 | 2 | 0 | 0 | 0 | 1 | 0 | 0 | 0 | 0 | 0 | 1 | 0 | 0 | 0 | 0 |
| **MIT 9215** | 0 | 2 | 0 | 0 | 0 | 1 | 0 | 0 | 0 | 0 | 0 | 1 | 0 | 0 | 0 | 0 |
| **MIT9312** | 2 | 2 | 0 | 2 | 1 | 1 | 0 | 2 | 0 | 0 | 0 | 1 | 0 | 0 | 0 | 0 |
| **NATL1A** | 0 | 6 | 0 | 0 | 2 | 0 | 1 | 0 | 0 | 0 | 0 | 0 | 0 | 0 | 0 | 0 |
| **NATL2A** | 1 | 0 | 0 | 0 | 0 | 0 | 0 | 0 | 0 | 0 | 0 | 0 | 0 | 0 | 0 | 0 |
| **PCC 6301** | 0 | 2 | 0 | 0 | 2 | 0 | 0 | 0 | 0 | 0 | 0 | 0 | 0 | 0 | 0 | 0 |
| **CC9311** | 1 | 7 | 0 | 1 | 1 | 0 | 0 | 2 | 0 | 1 | 0 | 1 | 0 | 0 | 0 | 0 |
| **CC9605** | 7 | 2 | 2 | 4 | 0 | 0 | 2 | 0 | 1 | 0 | 0 | 13 | 0 | 2 | 0 | 0 |
| **RCC307** | 0 | 5 | 0 | 0 | 0 | 0 | 0 | 1 | 0 | 0 | 0 | 0 | 0 | 0 | 0 | 0 |
| **WH7803** | 0 | 1 | 1 | 0 | 0 | 0 | 0 | 8 | 0 | 0 | 0 | 0 | 0 | 0 | 0 | 1 |
| **PCC6803** | 3 | 5 | 0 | 1 | 2 | 0 | 1 | 8 | 3 | 0 | 0 | 0 | 0 | 0 | 0 | 0 |
| **“D. ecotype”** | 0 | 1 | 0 | 4 | 1 | 4 | 1 | 12 | 4 | 8 | 0 | 10 | 0 | 0 | 1 | 0 |
| **DSM 3043** | 0 | 0 | 0 | 1 | 0 | 1 | 1 | 3 | 0 | 0 | 0 | 2 | 0 | 0 | 0 | 0 |
| **34H** | 0 | 0 | 0 | 3 | 0 | 1 | 0 | 0 | 0 | 0 | 0 | 2 | 0 | 0 | 0 | 0 |
| **L2TR** | 0 | 1 | 0 | 8 | 1 | 0 | 0 | 5 | 1 | 0 | 1 | 7 | 0 | 0 | 0 | 1 |
| **VT8** | 0 | 0 | 0 | 6 | 4 | 4 | 0 | 6 | 1 | 1 | 2 | 12 | 0 | 1 | 2 | 1 |
| **MWYL1** | 0 | 1 | 0 | 1 | 1 | 1 | 0 | 0 | 1 | 0 | 0 | 4 | 0 | 0 | 2 | 0 |
| **ATCC 19707** | 0 | 0 | 0 | 1 | 1 | 4 | 2 | 0 | 2 | 0 | 1 | 6 | 0 | 2 | 3 | 1 |
| **T6c** | 0 | 0 | 0 | 3 | 0 | 1 | 0 | 2 | 1 | 0 | 0 | 27 | 0 | 0 | 0 | 0 |
| **TAC125** | 0 | 2 | 0 | 0 | 0 | 1 | 2 | 5 | 2 | 2 | 1 | 4 | 0 | 0 | 0 | 0 |
| **273-4** | 0 | 2 | 0 | 4 | 2 | 1 | 0 | 2 | 0 | 0 | 0 | 11 | 0 | 0 | 0 | 0 |
| **K5** | 0 | 4 | 0 | 0 | 2 | 11 | 0 | 2 | 0 | 1 | 0 | 8 | 0 | 0 | 0 | 0 |
| **Prwf-1** | 0 | 3 | 0 | 0 | 6 | 3 | 0 | 8 | 2 | 0 | 0 | 13 | 0 | 0 | 0 | 0 |
| **OS155** | 0 | 0 | 26 | 3 | 12 | 1 | 3 | 3 | 4 | 9 | 1 | 31 | 0 | 0 | 0 | 0 |
| **OS217** | 0 | 4 | 0 | 2 | 2 | 1 | 1 | 1 | 0 | 1 | 0 | 3 | 0 | 0 | 0 | 0 |
| **MR-4** | 0 | 2 | 0 | 1 | 5 | 3 | 1 | 5 | 0 | 0 | 2 | 11 | 0 | 0 | 0 | 0 |
| **MR-7** | 0 | 2 | 0 | 2 | 2 | 2 | 0 | 2 | 1 | 0 | 0 | 5 | 0 | 0 | 0 | 0 |
| **O395** | 0 | 0 | 29 | 1 | 2 | 0 | 1 | 5 | 0 | 1 | 0 | 9 | 0 | 19 | 8 | 1 |
| **BAL3** | 0 | 5 | 0 | 1 | 0 | 0 | 0 | 6 | 0 | 0 | 2 | 5 | 0 | 0 | 1 | 1 |
| **HTCC1062** | 0 | 0 | 0 | 1 | 0 | 0 | 0 | 2 | 0 | 0 | 0 | 1 | 0 | 0 | 0 | 0 |
| **CCS1** | 0 | 0 | 0 | 0 | 0 | 0 | 0 | 2 | 4 | 0 | 0 | 11 | 0 | 0 | 0 | 1 |
| **Nb-255** | 0 | 5 | 0 | 1 | 1 | 4 | 0 | 3 | 1 | 0 | 3 | 30 | 0 | 0 | 1 | 0 |
| **ATCC17025** | 0 | 5 | 0 | 2 | 0 | 0 | 2 | 9 | 3 | 3 | 0 | 33 | 0 | 0 | 1 | 0 |
| **KD131** | 0 | 1 | 3 | 2 | 1 | 0 | 0 | 5 | 0 | 0 | 0 | 2 | 7 | 0 | 0 | 0 |
| **Och 114** | 0 | 0 | 0 | 1 | 0 | 10 | 1 | 5 | 3 | 21 | 0 | 8 | 0 | 0 | 0 | 0 |
| **CCS2** | 0 | 3 | 1 | 0 | 1 | 0 | 0 | 5 | 1 | 0 | 0 | 5 | 0 | 0 | 0 | 0 |
| **MED193** | 0 | 4 | 0 | 3 | 0 | 3 | 0 | 0 | 0 | 11 | 0 | 4 | 0 | 0 | 0 | 0 |
| **DSS-3** | 0 | 4 | 18 | 5 | 0 | 8 | 2 | 0 | 0 | 0 | 0 | 8 | 0 | 0 | 0 | 1 |
| **TM1040** | 0 | 1 | 0 | 1 | 0 | 0 | 0 | 0 | 0 | 0 | 0 | 12 | 0 | 0 | 0 | 0 |
| **TrichCH4B** | 0 | 0 | 0 | 2 | 1 | 2 | 2 | 5 | 2 | 0 | 1 | 3 | 0 | 0 | 0 | 1 |
| **RW1** | 0 | 0 | 5 | 4 | 2 | 0 | 2 | 4 | 0 | 0 | 0 | 12 | 0 | 0 | 0 | 0 |
| **EE36** | 0 | 0 | 32 | 3 | 0 | 1 | 4 | 1 | 0 | 0 | 0 | 7 | 0 | 0 | 0 | 0 |
| **R2A62** | 0 | 3 | 3 | 3 | 1 | 2 | 0 | 4 | 2 | 0 | 1 | 10 | 0 | 0 | 0 | 7 |
| **BAL3** | 0 | 5 | 0 | 1 | 0 | 0 | 0 | 6 | 0 | 0 | 2 | 5 | 0 | 0 | 1 | 1 |
| **M8** | 0 | 2 | 0 | 0 | 1 | 0 | 2 | 2 | 0 | 1 | 0 | 6 | 0 | 0 | 2 | 0 |
| **HTCC2559** | 0 | 0 | 0 | 1 | 0 | 1 | 0 | 3 | 0 | 0 | 1 | 1 | 0 | 0 | 0 | 0 |
| **ATCC 33406** | 0 | 0 | 0 | 3 | 0 | 5 | 0 | 9 | 0 | 1 | 0 | 2 | 0 | 0 | 0 | 0 |
| **MED134** | 0 | 0 | 0 | 0 | 0 | 0 | 0 | 5 | 0 | 0 | 1 | 2 | 0 | 0 | 0 | 0 |
| **BAL38** | 0 | 0 | 5 | 0 | 0 | 0 | 2 | 0 | 0 | 0 | 0 | 0 | 0 | 0 | 0 | 0 |
| **UW101** | 0 | 0 | 5 | 7 | 0 | 0 | 5 | 3 | 2 | 0 | 1 | 23 | 0 | 2 | 0 | 0 |
| **KT0803** | 0 | 3 | 5 | 2 | 0 | 0 | 2 | 2 | 0 | 0 | 0 | 0 | 0 | 0 | 3 | 0 |
| **OT-1** | 0 | 0 | 5 | 0 | 0 | 5 | 2 | 0 | 1 | 0 | 1 | 1 | 0 | 0 | 3 | 0 |
| **MED217** | 0 | 0 | 0 | 0 | 0 | 0 | 0 | 1 | 0 | 0 | 0 | 3 | 4 | 0 | 0 | 0 |
| **23-P** | 0 | 1 | 4 | 0 | 0 | 0 | 2 | 0 | 0 | 0 | 0 | 0 | 0 | 0 | 0 | 0 |
| **MED152** | 0 | 0 | 0 | 0 | 0 | 0 | 0 | 0 | 0 | 0 | 0 | 0 | 0 | 0 | 0 | 0 |
| **HTCC2501** | 0 | 4 | 5 | 0 | 0 | 5 | 2 | 5 | 1 | 0 | 1 | 1 | 0 | 0 | 0 | 0 |

^a^ The complete bacterial name is shown in Table 3SM in the same order. Here only the name of the strain is shown.

^b^ Each of the 16 biological categories are detailed in Material & Methods and placed in the same order as in Figure 10.
